# Nicotinamide adenine dinucleotide kinase promotes lymph node metastasis of NSCLC via activating ID1 expression through BMP pathway

**DOI:** 10.7150/ijbs.84322

**Published:** 2023-06-19

**Authors:** Zimei Zeng, Jie Gao, Tao Chen, Ziyu Zhang, Mengwei Li, Qi Fan, Guoqian Liu, Xuebing Li, Zhi Li, Chenxi Zhong, Feng Yao, Lunquan Sun, Yuezhen Deng, Min Li

**Affiliations:** 1 Department of Oncology, Xiangya Cancer Center, Xiangya Hospital, Central South University, Hunan Province, Changsha 410008, China.; 2 Key Laboratory of Molecular Radiation Oncology Hunan Province, Hunan Province, Changsha 410008, China.; 3 Hunan International Science and Technology Collaboration Base of Precision Medicine for Cancer, Hunan Province, Changsha 410008, China.; 4 State Key Laboratory of Respiratory Diseases at People's Hospital of Yangjiang, Yangjiang, Guangdong 529500, China.; 5 Yangjiang Key Laboratory of Respiratory Disease, People's Hospital of Yangjiang, Yangjiang, Guangdong 529500, China.; 6 Department of Respiratory Medicine, National Key Clinical Specialty, Branch of National Clinical Research Center for Respiratory Disease, Xiangya Hospital, Central South University, Changsha, China.; 7 Xiangya Lung Cancer Center, Xiangya Hospital, Central South University, Changsha, China.; 8 Center of Respiratory Medicine, Xiangya Hospital, Central South University, Changsha, Hunan, China.; 9 Clinical Research Center for Respiratory Diseases in Hunan Province, Changsha, China.; 10 National Clinical Research Center for Geriatric Disorders, Changsha, China.; 11 Department of Thoracic Surgery, Shanghai Chest Hospital, Shanghai Jiao Tong University School of Medicine, 200030, Shanghai, China.; 12 Tianjin Key Laboratory of Lung Cancer Metastasis and Tumor Microenvironment, Tianjin Lung Cancer Institute, Department of Lung Cancer Surgery, Tianjin Medical University General Hospital, Tianjin, China.

**Keywords:** Lymph node metastasis, Non-small cell lung cancer (NSCLC), NAD Kinase, BMPs signalling, Inhibitor of differentiation 1.

## Abstract

Metastasis is a significant cause of high mortality in lung cancer. Lymph node (LN) metastasis is the most common metastatic pathway in non-small cell lung cancer and the most crucial factor affecting the prognosis of NSCLC. Nevertheless, the molecular mechanism underlying metastasis is unknown. We demonstrated that higher NADK expression suggests worsened survival prognosis, and NADK expression positively correlates with the lymph node metastasis rate and TNM and AJCC stages in NSCLC patients. Moreover, patients with LN metastasis show higher NADK expression than those without LN metastasis. NADK can promote NSCLC progression by enhancing the migration, invasion, lymph node metastasis and growth of NSCLC cells. Mechanistically, NADK inhibits the ubiquitination and degradation of BMPR1A by interacting with Smurf1, further activating the BMPs signalling pathway and promoting ID1 transcription. In conclusion, NADK may be a potential diagnostic indicator and a novel therapeutic target for metastatic NSCLC.

## Introduction

Globally, the incidence of lung cancer ranks first among 36 common cancers. It remains the leading cause of cancer-related deaths^1^. Non-small cell lung cancer (NSCLC) is the most common type of lung cancer on the basis of histological morphology^2^. Approximately 70% of patients with NSCLC are diagnosed with clinically advanced disease, indicated by invasive lymph node metastasis, which results in few surgical opportunities and unsatisfactory clinical outcomes^2^, ^3^.

**Figure 1:**
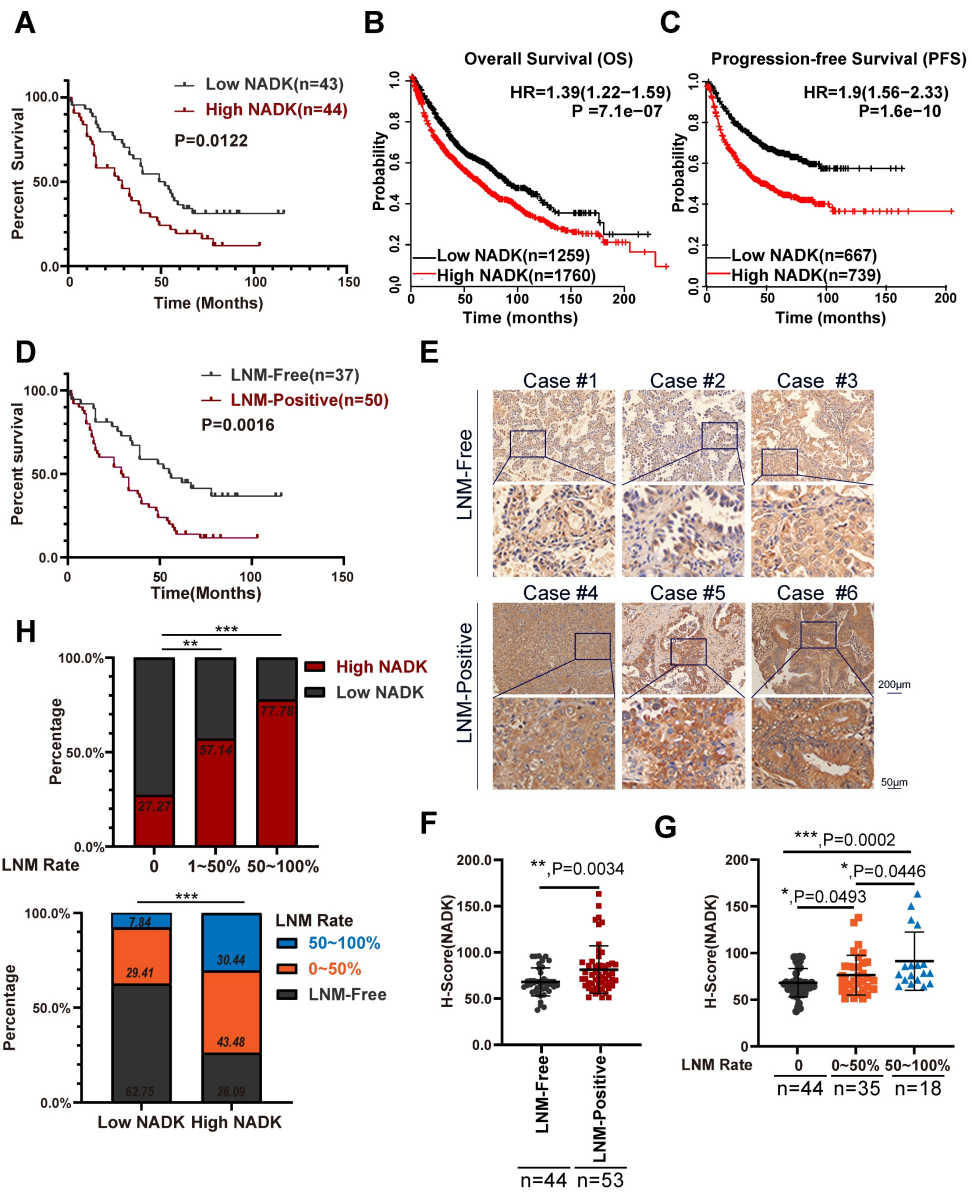
** NADK is highly expressed in NSCLC patients with LNM.** Figure** 1A.** Analysis of the correlation between the expression of NADK and the overall survival of 87 NSCLC cases. **1B-C.** The Kaplan-Meier Plotter database was used to analyse the correlation between the expression of NADK and the overall survival (**1B**) or progression-free survival (**1C**) of patients with NSCLC. The scale bars are shown. **1D**. The correlation between the LN metastasis status and the overall survival of 87 NSCLC cases was analyzed. **1E.** Representative IHC images showing the expression of NADK in 3 NSCLC tissues with positive LN metastasis (LNM-Positive) and 3 NSCLC tissues without LN metastasis (LNM-Free), respectively. **1F.** The NADK expression between LNM-positive patients and LNM-negative patients was analysed with unpaired *t* test. **1G.** The NADK expression among NSCLC patients without LN metastasis, LNM rate less than 50% and LNM rate higher than 50% was examined using unpaired *t* test. **1H.** The percentage of patient with high NADK expression in different LN metastasis groups (up). The percentage of patients with different LNM status in “high NADK” group and “low NADK” group, respectively. (down).

Many studies have addressed the mechanisms of tumor metastasis involving the bloodstream to distant organs, even though the majority of epithelial cancers first develop metastatic growth by spreading via lymphatic vessels to draining LNs (lymph nodes)^4^. The prevailing view suggests that lymphatic vessels play a mere passive role in tumor metastasis, serving as channels for tissue-invading tumor cells^4^, ^5^. Despite the obvious vital clinical role of LN metastasis, the mechanisms leading to tumor spread via lymphatic vessels remain to be explored. LN metastasis is the most common and major pathway of non-small cell lung cancer metastatic, largely affecting the staging and prognosis of NSCLC^6^. In recent years, in-depth studies have been carried out on LN metastasis in NSCLC for the purpose of improving the prognosis of patients with NSCLC and discovering the significance of LN metastasis in treatments for NSCLC. To date, studies have shown that patients with lymph node metastasis (LNM) present with shorter survival times than those without LNM, which has also been demonstrated in our study (Figure 1D). Even in Stage I NSCLC patients who undergo complete tumor resection, the incidence of postoperative recurrence or metastasis is as high as 21.7%, which shows a relationship with early lymph node metastasis 7. Studies have shown that patients with different areas of LNM, such as the hilar lung (N1) and mediastinum (N2), also have different prognoses. Bills et al. reviewed 1667 patients with clinical Stage I NSCLC who underwent anatomic lung resection and lymphadenectomy and found that the median overall survival time was 83.7 months for N0 patients, and it was 48.0 and 37.9 months for N1 and N2 patients, respectively^8^. Therefore, there is an urgent need to find new biomarkers for predicting lymph node metastasis in NSCLC patients and provide novel treatment strategies for NSCLC patients with lymph node metastasis.

The BMPs signalling pathway plays an inhibitory role in the initial stage of tumor development while promotes cell proliferation, cell stemness, invasion and metastasis in the late stages of tumor. BMP transduces signals through two highly conserved single-transmembrane serine/threonine kinase receptors called Type II receptors (BMPR2) and Type I receptors (BMPR1A/1B, or ALK3/6). The binding of BMP ligands (BMP-2, BMP-7, etc.) can induce the formation of heterotetrameric receptor complexes, resulting in the phosphorylation (activation) of Type I receptors by Type II receptors.

The activation of BMPR1A/1B phosphorylates Smad1/5. Phospho-Smad1/5 interacts with co-mediator Smad (co-Smad), Smad4, and is translocated to the nucleus, where it regulates and initiates the transcription of target genes. In addition to the its various functions in physiological processes, BMPs signalling plays a pleiotropic role in cancer progression. Previously, the activation of BMPs signalling was shown to promote bone metastasis of NSCLC cells^9^, while BMP signalling inhibition impaired NSCLC cell viability, growth and migration^10^. BMPR1A plays an initial role in BMP signalling activation, and it is ubiquitinated by the E3 ubiquitin ligase Smurf1 and thus degraded by the proteasome. In many tumor tissues, BMPR1A showed higher expression than nontumor tissues and was closely related to tumor growth and metastasis^11^-^13^. The inhibitors of BMP receptor kinase have been shown to exert effective antitumor effects on ovarian cancer and endometrial cancer 14-16. However, the mechanism underlying the action of highly expressed BMPR1A remains to be explored. In addition, the expression of BMPs is higher in cancer cells than in normal lung tissue, and promotes angiogenesis and metastasis in lung cancer.

Due to rampant growth and proliferation, cancer cells are stressed by hypoxia and mitochondrial damage, resulting in a continuous increase in the production of inherently reactive oxygen species (ROS), which enables cells to maintain a carcinogenic phenotype and drives tumor progression^16^, ^17^. Cells stimulated by increased ROS production become spindle shaped and undergo morphological changes, including reduced cell-cell adhesion and loss of polarity, which are characteristic of the mesenchymal phenotype, suggesting an underlying role for ROS in cancer spread^17^, ^18^. NADPH can scavenge ROS because it reduces glutathione, peroxiredoxin and the catalase tetramer. NADK is the rate-limiting enzyme in NADPH synthesis, mediating the conversion of NAD to NADP. NADP is then rapidly converted to NADPH via G6PD and malic enzymes (MEs). Studies have shown the plasticity of NADK activity. AKT-mediated phosphorylation of the N-terminal domain of NADK dampens NADK activity. Phosphorylated NADK undergoes a conformational change, which endows it with higher enzymatic activity, promoting the synthesis of NADP (the rate-limiting substrate in NADPH production)^19^. Lung cancer cells with phosphorylated NADK show improved growth ability. However, it has been reported that in pancreatic ductal adenocarcinoma, KRAS mutations promote the phosphorylation of NADK by PKC, which increases NADK activity and promotes the synthesis of NADPH, promoting the progression of pancreatic cancer^20^. In addition, the NADK I90F mutation has been found to exhibit higher enzymatic activity in pancreatic cancer, and by altering tumor redox homeostasis, it regulates pancreatic cancer progression^21^. Some inhibitors have been designed for attenuating NADK activity to a certain extent; for example, thionicotinamide (TN) and 6-amino nicotinamide are NADK inhibitors^22^. Many studies have shown that metabolic kinases are involved in nonmetabolic pathways^23^, but it is not yet clear whether NADK exhibits nonmetabolic functions.

In this study, we found that NADK expression was increased in NSCLC, and high expression of NADK predicted a worse survival prognosis. NADK expression positively correlated with lymph node metastasis and tumor stage in NSCLC patients. NADK promoted the proliferation, invasion and lymph node metastasis of NSCLC cells. This study explores the function and molecular mechanism of NADK in the progression of NSCLC.

## Results

### NADK is highly expressed in NSCLC patients with LNM

It has been reported that phosphorylation of NADK by AKT (p-NADK^S44/46^) upregulates the enzymatic activity of NADK, and p-NADK^S44/46^ shows a stronger proliferation-promoting function in lung cancer cells than that of wild-type NADK^19^. However, further studies are needed to understand the specific functions and mechanisms of wild-type NADK in NSCLC. We first measured the mRNA levels of NADK in lung cancer cell lines and lung cancer tissues. The expression level of NADK in lung cancer cells was higher than that in normal cells (Supplementary Fig. 1A). Analysing the mRNA levels of 56 paired lung cancer tissue and normal tissue showed that NADK expression was increased in tumor tissues compared with adjacent nontumor tissues (Supplementary Fig. 1B). Western blot experiments showed that NADK protein levels were significantly increased in lung cancer tissues (8/9) (Supplementary Fig. 1E). Immunohistochemistry (IHC) examination of lung cancer tissue arrays (containing 81 pairs of adjacent nontumor tissues and tumor tissues) revealed that the expression level of NADK protein was significantly increased in lung cancer tissues (Supplementary Figure 1C-D). Furthermore, high expression of NADK in NSCLC patients predicted a poor survival (Figure 1A), which was consistent with the data described in the Kaplan‒Meier plotter database (Figure 1B-C) and GEPIA database (Supplementary Figure 1E).

As mentioned previously, LN metastasis indicates poor survival in NSCLC patients (Figure 1D). In our study, NADK expression showed positive correlations with the TNM and AJCC stages of NSCLC patients, especially lymph node metastasis (Table 1). NSCLC patients with LN metastasis showed higher NADK expression than patients without LNM (Figure 1E-F), and a higher LNM rate was accompanied by higher NADK expression (Figure 1G). Moreover, a higher positive rate of lymph node metastasis indicates a higher probability of expressing a higher level of NADK (Figure 1H). Additionally, the lymph node metastasis rate was higher in patients with high NADK expression (Figure 1H). In conclusion, these results suggest that NADK may play a potential vital role in the lymph node metastasis of NSCLC.

### NADK promotes migration, invasion and growth of NSCLC cells

**Figure 2:**
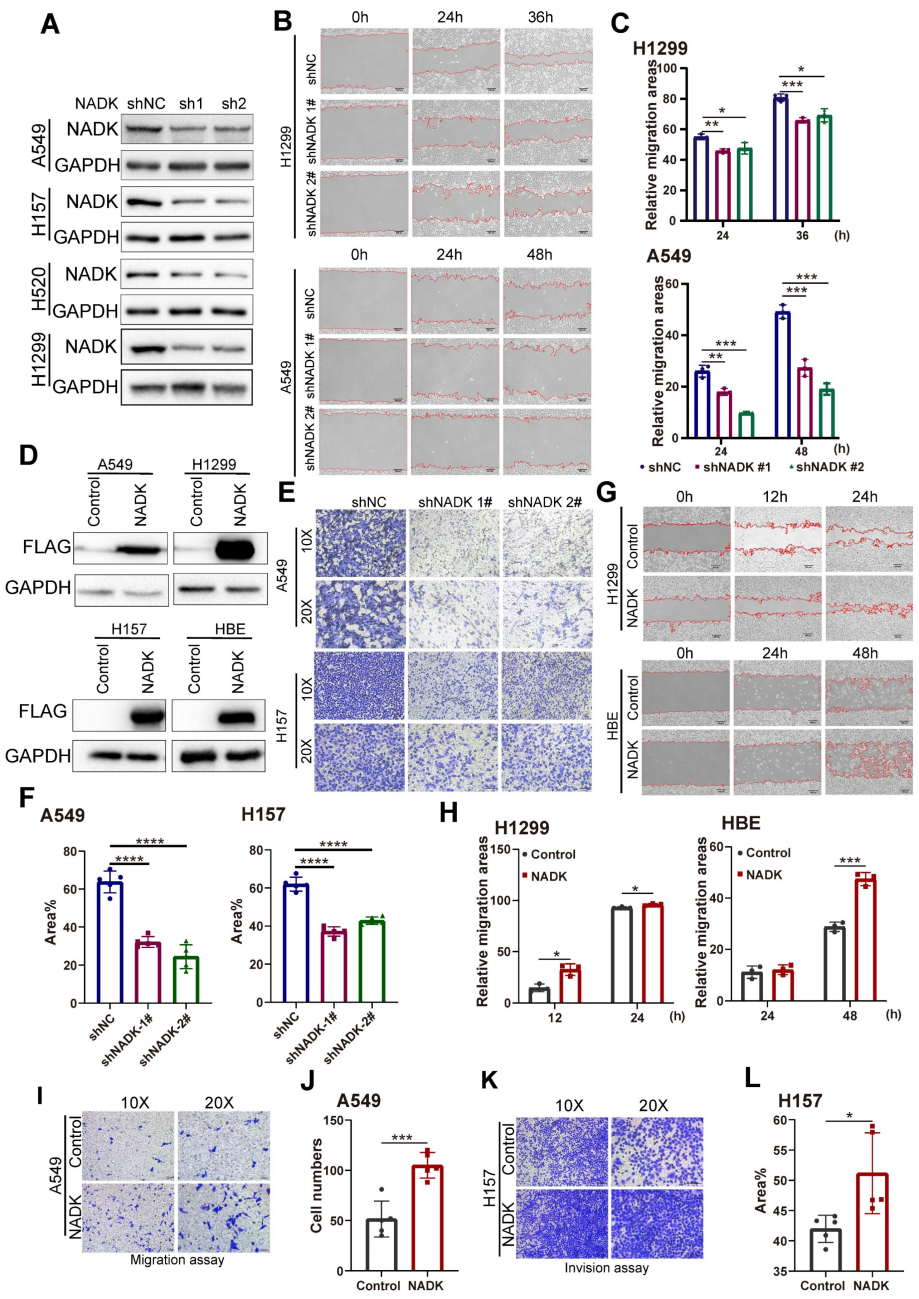
** The migration, invasion of NSCLC cells were promoted by NADK.** Figure **2A**. NADK expression was knocked down in A549, H157, H520 and H1299 cells. The cells were infected with the shNADK lentivirus and control lentivirus (shNC). After the selection with puromycin, the resistant cells were pooled, and Western blotting was used to detect the protein level of NADK. **2B-C**. Wound-healing assay examined the migration ability of NSCLC cells (H1299, A549) with NADK knockdown(**2B**), relative migration areas were quantified and analysed (**2C**). **2D.** NADK was overexpressed in normal HBE cells and NSCLC cells (A549, H1299 and H157). Cells were infected with lentivirus overexpressing Flag-NADK or control lentivirus, and selected with puromycin. Western blot was used to detect the overexpression of NADK with anti-Flag antibody. **2E-F.** Invasion assay using the Transwell was conducted to quantify and analyse the effect of NADK knockdown on the invasion of A549 and H157 cells.** 2G-H.** Wound-healing assay was performed to examine the effect of NADK on the migration of H1299 and HBE cells (**2G**), and relative migration areas were analysed (**2H**).** 2I-J.** Migration assay using the Transwell was conducted to examine the effect of NADK expression on the motility of A549 cells (**2I**). The migratory cells were quantified and analysed (**2J**). **2K-L.** Invasion assay using the Transwell was preformed to detect the effect of NADK over-expression on the invasion of H157 cells (**2K**). The invasive areas were quantified and analysed (**2L**). The scale bars were indicated. *, *P*<0.05; ***, *P*<0.001; ****, *P*<0.0001.

To identify the function of NADK in the progression of NSCLC, NADK was knocked down in four NSCLC cell lines (the A549, H157 H520 and H1299 cell lines) (Figure 2A). Wound-healing assay showed knockdown of NADK attenuated the migration of NSCLC cells (Figure 2B-C). In addition, Transwell assays showed that down-regulation of NADK significantly reduced the invasion of NSCLC cells (Figure 2E-F). Moreover, a CCK8 assay showed that interfering with NADK expression inhibited NSCLC cell proliferation (Supplementary Fig. 2A). Similarly, knockdown of NADK impaired the anchorage-independent growth of NSCLC cells (Supplementary Fig. 2C-D). In summary, knockdown of NADK inhibited the proliferation, anchorage-independent growth, and invasion of NSCLC cells *in vitro*.

To validate the findings described above, NADK was overexpressed in normal human bronchial epithelial cells (HBE) and three NSCLC cell lines (the A549, H157 and H1299 cell lines) (Figure 2D). A wound-healing assay showed that overexpressed NADK promoted NSCLC cell migration (Figure 2G-H). In addition, transwell assay revealed the enhanced ability of migration and invasion in NADK-overexpressing NSCLC cells (Figure 2I-L). Moreover, a CCK8 assay showed that overexpressed NADK promoted the proliferation of A549, H1299 and HBE cells (Supplementary Fig. 2B), and a soft agar assay indicated that the overexpression of NADK promoted the anchorage-independent growth of HBE, A549 and H1299 cells (Supplementary Fig. 2E-F). Herein, we demonstrated that NADK promotes the migration, invasion and growth of NSCLC cell* in vitro*.

### NADK promotes lymph node metastasis and growth of NSCLC cells *in vivo*

**Figure 3:**
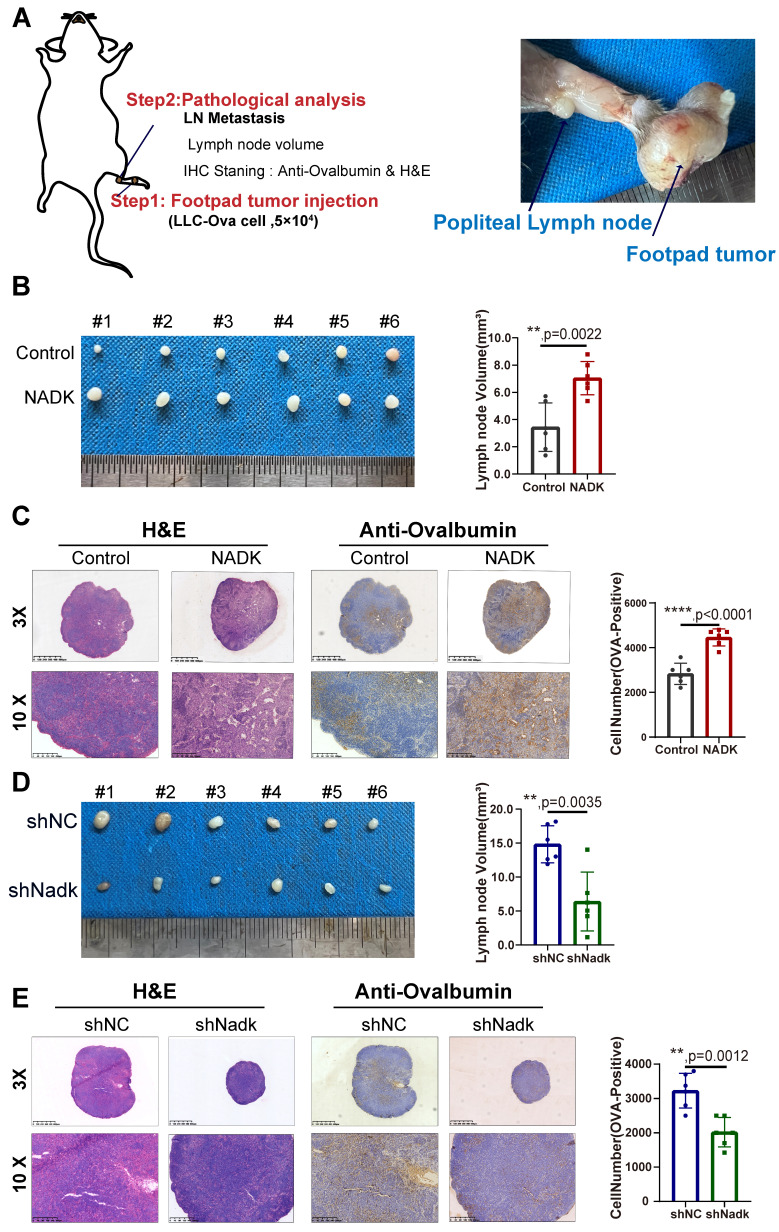
** NADK promotes the lymph node metastasis of NSCLC cells.** Figure **3A.** The schematic illustration of LN metastasis model preformed on C57BL/6 mice.** 3B.** LN metastasis model was conducted on C57BL/6 mice with LLC-NADK overexpression cells, popliteal lymph nodes were isolated at the end of the experiment, and LN volume was measured and analysed. **3C.** IHC staining and HE staining were performed on lymph node tissues in **3B** with anti-Ovalbumin antibody. Ovalbumin positive cells were counted and analysed. Figure **3D.** LN metastasis model was performed on C57BL/6J mice using LLC cells with knockdown of NADK. Popliteal lymph nodes were isolated at the end of the experiment, and LN volume was measured and analysed. **3E.** Lymph node tissues in **3D** were examined using IHC staining with anti-Ovalbumin antibody or H&E staining. Cells expressing Ovalbumin were counted and analysed. The scale bars were indicated. **, *P*<0.01.

Considering that NADK expression was positively correlated with the LN metastasis, we further evaluated the function of NADK in LN metastasis in C57BL/6J mouse models of LN metastasis. The LLC-Ova cell line expressing the chicken protein Ovalbumin was constructed, which acted as a marker of tumor cells in LN metastasis. In these cells, Ovalbumin functioned as a tumor antigen that was detected by IHC staining using an anti-Ovalbumin antibody. We injected LLC-Ova control cells and NADK-overexpressing cells into the footpad of C57/BL6 mice and collected the popliteal lymph nodes from the mice 4 weeks later (Figure 3A). The LN volume revealed higher metastatic potential of NADK-overexpressing LLC cells *in vivo* (Figure 3B), which were consistent with the H&E and IHC staining results (Figure 3C). In addition, knockdown of NADK expression resulted in a smaller lymph node volume and fewer metastatic cells (Figure 3D-E). In conclusion, NADK promotes the LN metastasis of NSCLC cells.

Next, to verify the function of NADK in promoting the tumor growth, we first subcutaneously inoculated LLC cells overexpressing NADK into C57/BL6 mice. Compared with the control group, NADK overexpression led to larger tumor size, an increased tumor growth rate (Supplementary Fig. 3A-B), and higher tumor weight (Supplementary Fig. 3C). Ki67, a cell proliferation index, was measured by immunohistochemical staining, and the intensity of the staining was scored. The results of IHC staining for Ki67 further demonstrated a growth-promoting role for NADK (Supplementary Fig. 3D, 3E). Similarly, knocking down NADK reduced the tumorigenic ability of the LLC cells *in vivo* (Supplementary Fig. 3F-H) as well as the Ki67 expression (Supplementary Fig. 3I-J). Taken together, these results demonstrated that NADK promotes the tumorigenesis and LN metastasis *in vivo*.

### NADK inhibits ubiquitination and degradation of BMPR1A by interacting with Smurf1

**Figure 4:**
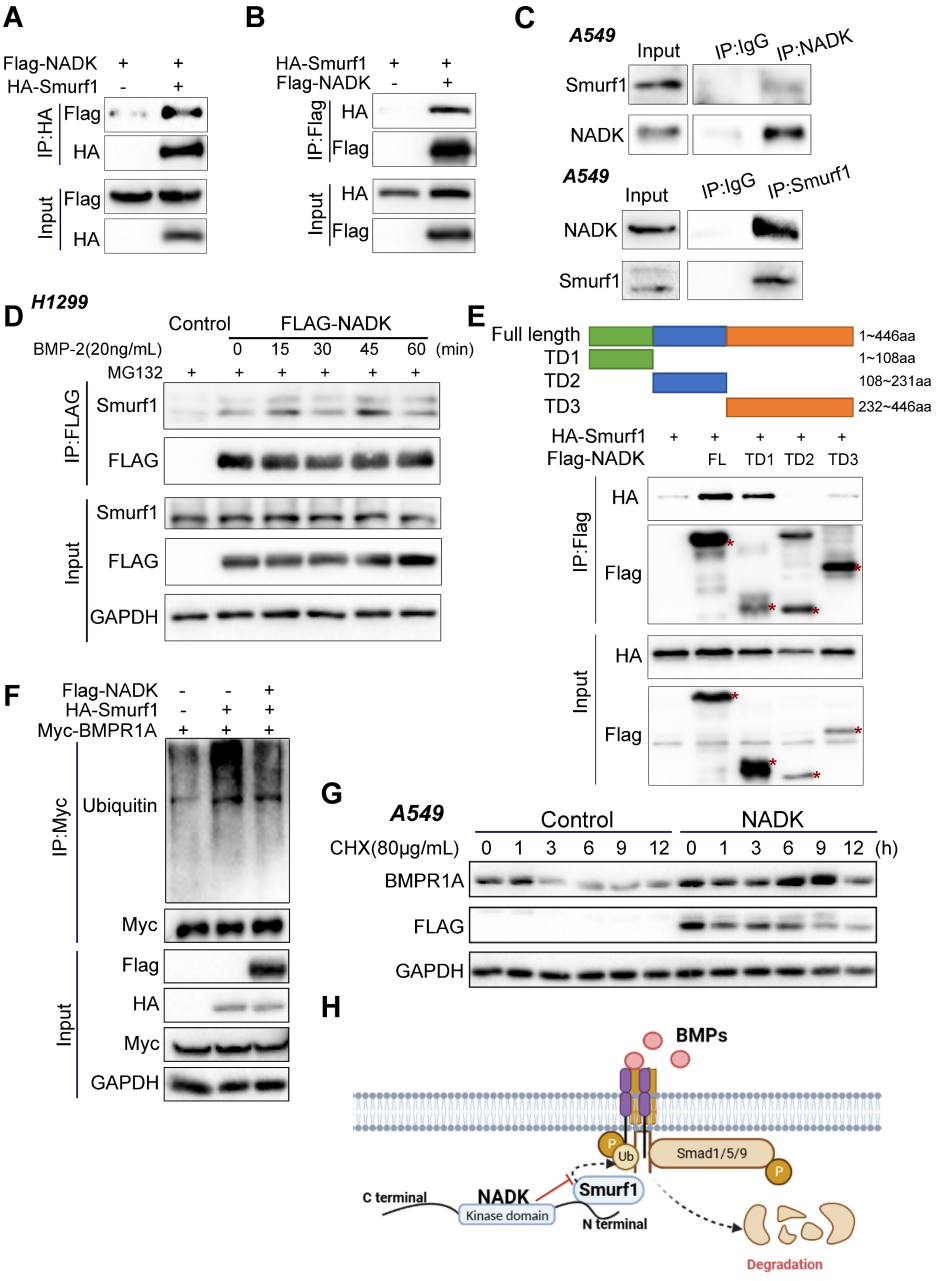
** NADK inhibits the ubiquitination and degradation of BMPR1A by interacting with Smurf1. Figure 4A-B.** Co-IP was performed to detect the interaction between the exogenously expressed Flag-NADK and HA-Smurf1. **4C.** The interaction between endogenously expressed NADK and Smurf1 was detected by coimmunoprecipitation in A549 cells. **4D.** The interaction between NADK and Smurf1 was induced by BMP2. Cells treated with BMP-2 recombined protein were harvested at different time points for Co-IP.** 4E.** Co-IP was used to determine the binding domain of NADK for the interaction with Smurf1. The full length NADK and truncation domain were shown. Smurf1 and NADK or its truncation mutants were co-transfected into HEK293T cells. After 36 hours, Co-IP was performed. **4F.** Ubiquitin assay were performed to detect the effect of NADK on the ubiquitination of BMPR1A by Smurf1. **4G.** The effect of overexpression of NADK in A549 cells on the half-life of BMPR1A protein level was detected. Cells were treated with CHX (80µg/ml) for different periods of time, and the BMPR1A protein was measured by western blot. **4H.** Graphic summary. N-terminal of NADK interacted with Smurf1, and impaired the degradation of BMPR1A.

To identify the underlying molecular mechanism through which NADK promoted the LN metastasis of NSCLC, we performed RNA-seq using the NADK-overexpressing A549 cells and control cells. Differentially expressed genes (DEGs) with an expression fold change higher than 1.5 enriched in KEGG pathways were identified with iDEP.96 (bioinformatics.sdstate.edu). The 50 KEGG pathways influenced most profoundly by NADK were listed in Supplementary Table 3. The TGFβ signalling pathway was found to be upregulated in NADK-overexpressing A549 cells. Considering the ligands of TGFβ and BMP belong to the same family, and therefore we focused on both BMP and TGFβ signalling (Supplementary Fig. 4A), which have been reported to exert universal effects on tumor metastasis^24^, ^25^. We further explored the possibility that NADK regulates metastasis by interacting with the components in the BMP and TGFβ signalling pathway. Co-IP was performed to identify proteins that possibly interact with NADK (Supplementary Fig. 4B). Fortunately, we identified an interaction between NADK and the ubiquitination regulatory enzyme Smurf1 (Figure 4A-B), which ubiquitinates Smad1/Smad5 or BMPR1 and promotes them to degrade, which was consistent with the results from the RNA-seq analysis that NADK showed the most profound effects on BMP signalling. We further verified the interaction between endogenous NADK and Smurf1 through IP (Figure 4C). Moreover, the treatment of BMP enhanced the binding of NADK and Smurf1 (Figure 4D). We therefore mapped the binding region for Smurf1 in NADK protein. The NADK protein was divided into three parts: TD1 (1-108, kinase-inhibiting domain), TD2 (108-231, ATP-binding domain), and TD3 (232-446), and TD1 was found to mediated the interaction between NADK and Smurf1 using the immunoprecipitation assay (Figure 4E). We then examined the effects of NADK expression on the ubiquitination level of BMPR1A. As shown in Figure 4F, NADK was found to inhibit the ubiquitination of BMPR1A by Smurf1 (Figure 4F). Furthermore, the effects of NADK expression on the half-life of BMPR1A were determined. The result showed that overexpression of NADK prolonged the half-life of BMPR1A, indicated NADK stabilized BMPR1A protein (Figure 4G). In summary, NADK stabilizes BMPR1A protein by inhibiting the ubiquitination-mediated degradation of BMPR1A (Figure 4H).

### NADK activates BMPs signalling pathway and the transcription of ID1 in lung cancer cells

**Figure 5:**
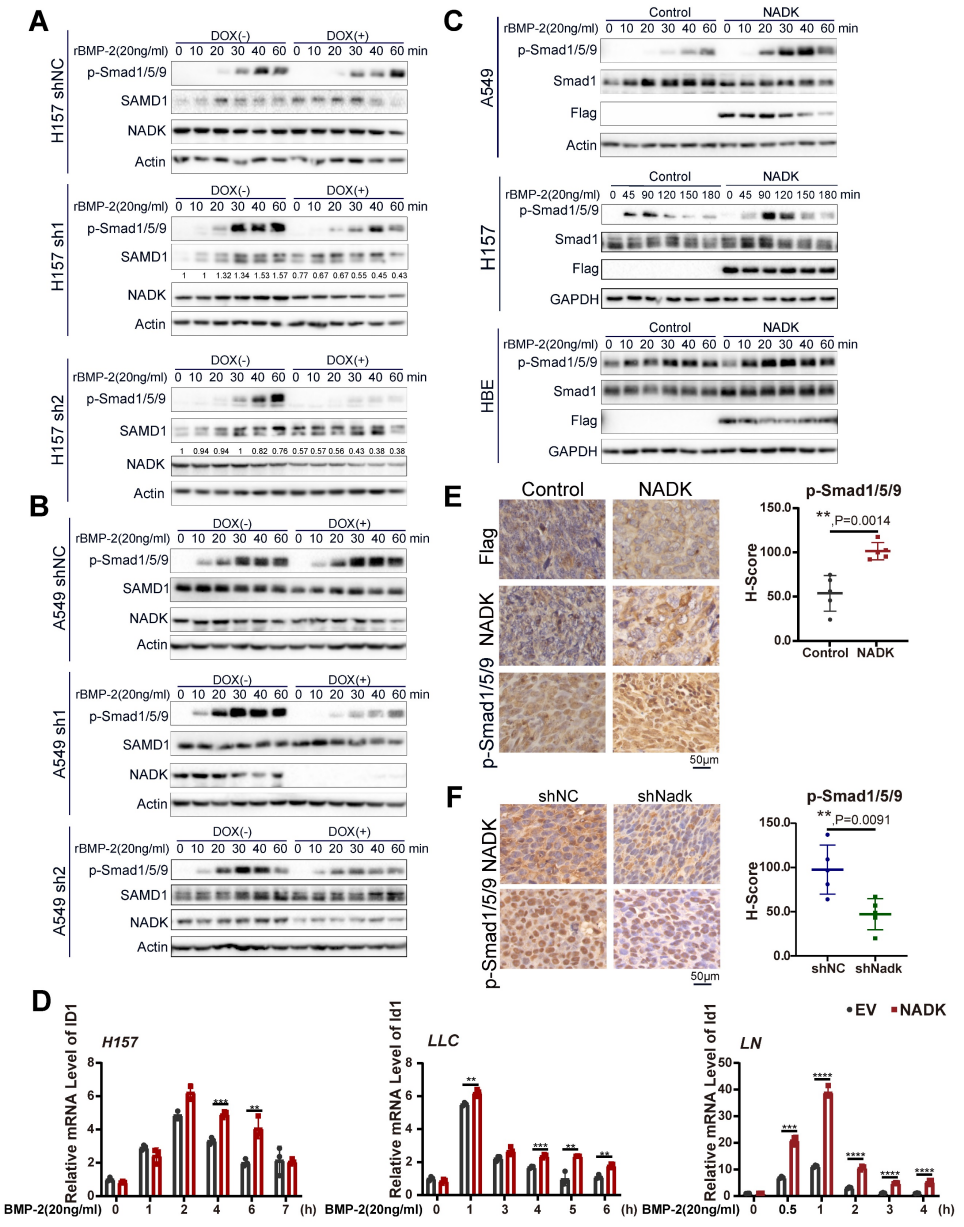
** Activation of BMP signalling pathway by NADK in lung cancer cells.** Figure **5A-5B.** Western blotting was used to evaluate the effect of NADK knockdown on the phosphorylation of Smad1/5/9 in H157 and A549 cells. Tet-on system was used to knock down NADK upon the administration of DOX. **5C.** Phosphorylation levels of Smad1/5/9 were examined in A549, H157, HBE cells with the overexpression of NADK.** 5D.** QPCR assays were used to detect the effects of NADK over-expression on the induction of ID1 mRNA level upon the treatment of BMP-2. **5E-F.** The levels of Phosphorylated Smad1/5/9 in the xenografts derived from the cells with overexpression or knockdown of NADK were examined by IHC and analysed. The scale bars were indicated. **, *P*<0.01; ***, *P*<0.001; ****, *P*<0.0001.

BMPs signalling has been found to enhance the bone metastasis of NSCLC cells 9, and its inhibition reduced the viability, growth and migration of NSCLC cell 10. Among all BMP ligands, BMP-2 has been reported to be the most highly expressed in NSCLC tissues 26. Therefore, BMP-2 was chosen as the ligand used to activate BMP signalling in this study. To examine the function of NADK in mediating BMP signalling activation, we first measured the phosphorylation of Smad1/5/9 (phospho-Smad1 (Ser463/465)/Smad5 (Ser463/465)/Smad9 (Ser465/467)). NADK was knocked down with a Tet-on system, and NADK knockdown inhibited the induction of p-Smad1/5/9 in both H157 and A549 cells upon the treatment of BMP2 (Figure 5A-B). In addition, NADK did not cooperate with TGFβ to increase the levels of phospho-Smad2, further indicating NADK exerted few effects on TGFβ pathway (Supplementary Figure 5A).

**Figure 6:**
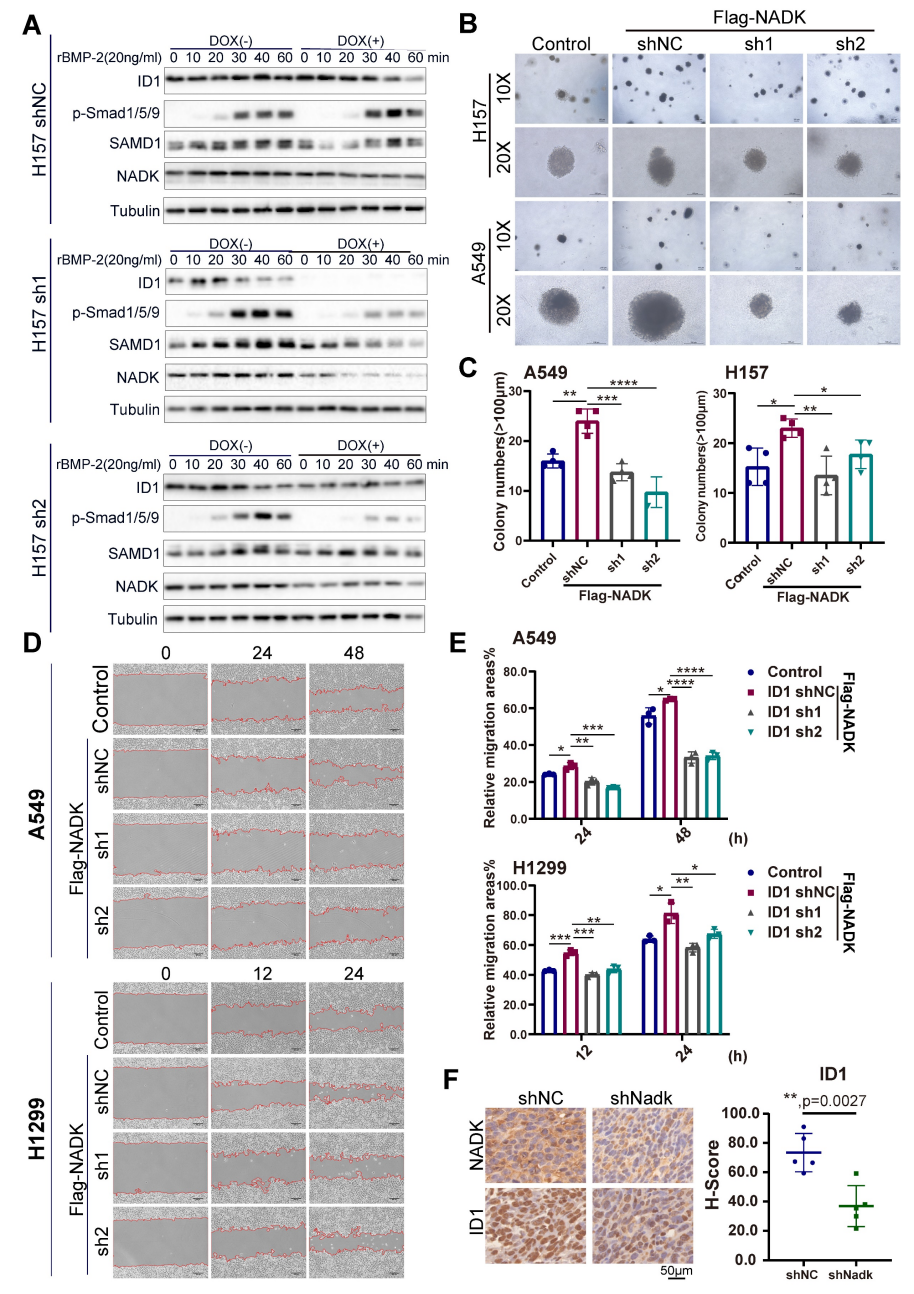
** Knockdown of ID1 attenuates the functions of NADK in NSCLC cells.** Figure **6A.** Western blotting was performed to examine the effect of NADK knockdown on the induction of ID1 protein by BMP-2. **6B-C.** Soft agar assay was used to evaluate the effect of ID1 knockdown on the anchor-independent growth of A549 and H157 cells promoted by NADK overexpression. Colony number was counted and analysed (**6C**). **6D-6E.** Wound-healing assay was performed to detect the effect of ID1 knockdown on the migration of A549 and H1299 cells promoted by NADK. Relative migration areas were measured and analysed (**6E**). **6F.** IHC staining of ID1 in xenografts formed by the cells with the knockdown of NADK. The expression of ID1 was scored and analysed. The scale bars were indicated. *, *P*<0.05; **, P<0.01; ***, *P*<0.001; ****, *P*<0.0001.

However, NADK overexpression enhanced the activation of BMP signalling in NSCLC cells (Figure 5C, Supplementary Figure 5B). The inhibitor of DNA binding (ID) is transcription target gene of the BMP signalling in lung cancer cells^27^, ^28^ and promotes lung cancer cell metastasis^29^. Moreover, RNA-seq analysis revealed the up-regulation of IDs in NADK-overexpressing cells. Therefore, we examined whether NADK promoted the expression of ID1 through BMP signalling. It was found that overexpression of NADK further cooperated with BMP2 to induce the ID1 mRNA in H157, LLC and LN cells (Figure 5D), further suggesting that NADK activated BMP pathway. Moreover, an increase in the phosphorylation level of Smad1/5/9 was observed in the xenografts derived from the cells with the overexpression of NADK (Figure 5E), and the phosphorylation level of Smad1/5/9 decreased in the xenografts derived from the cells with the knockdown of NADK (Figure 5F). Similarly, knockdown of NADK impaired the induction of ID1 protein upon the treatment of BMP-2 in H157 cells (Figure 6A). In addition, a positive correlation between the mRNA expression levels of NADK and ID1 was found in TCGA and GTEx databases (Supplementary Fig. 5C).

Then, we further investigated whether ID1 mediated the biological function of NADK in the progression of NSCLC. ID1 was knocked down by shRNA in NADK-overexpressing H1299, A549, H157 and LN cells (Supplementary Fig. 5D), and the downregulation of ID1 in NSCLC cells impaired the anchorage-independent growth (Figure 6B-C) and migration (Figure 6D-E, Supplementary Fig. 5E) driven by the overexpression of NADK. Consistently, the increase in ID1 expression was observed in the xenograft derived from the cells with NADK overexpression (Supplementary Fig. 5F), and the reduction of ID1 expression was found in the xenograft formed by the cells with the knockdown of NADK (Figure 6F).

**Figure 7:**
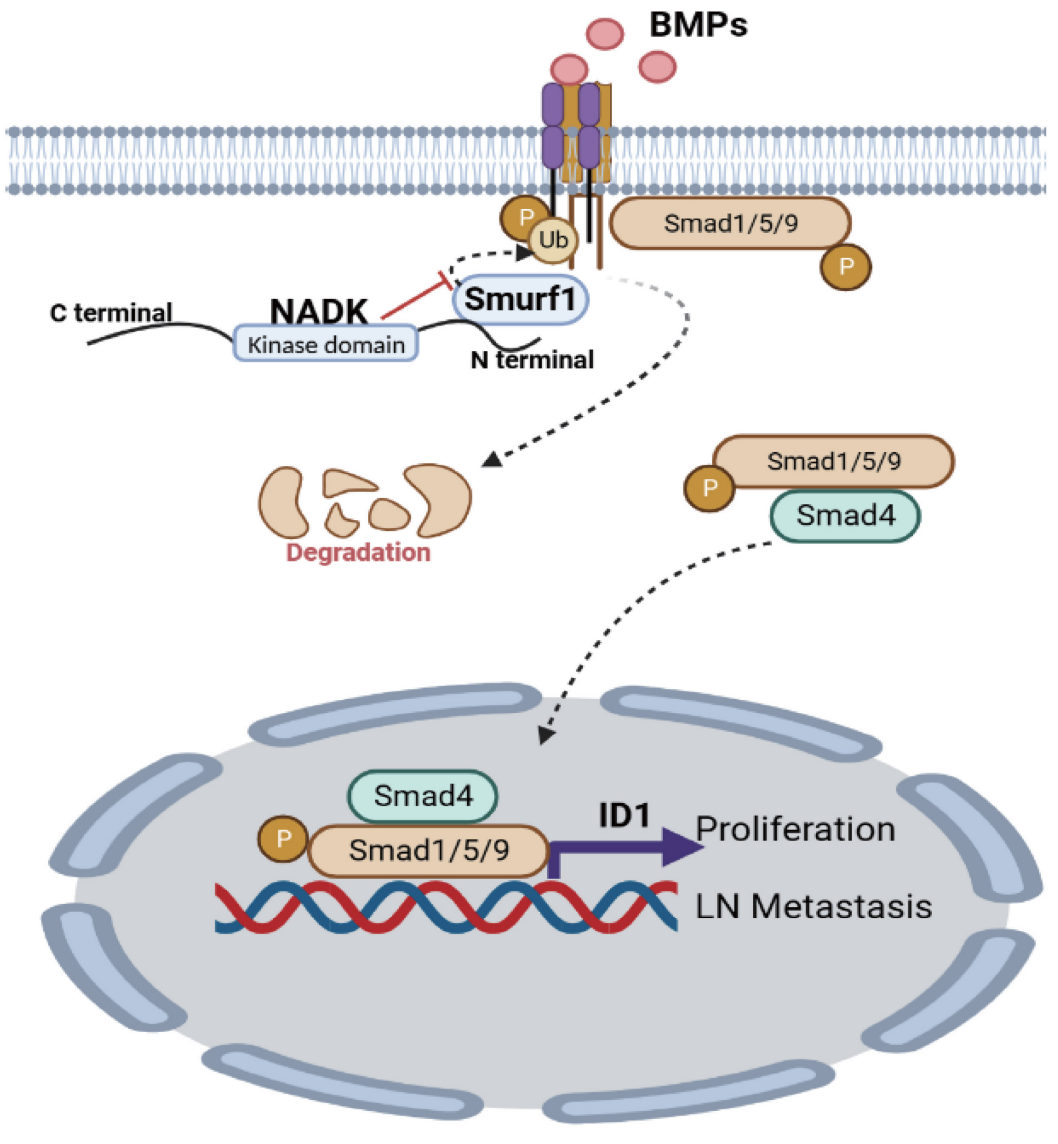
** Graphic abstract.** NADK activates BMPs/ID1 signalling by binding Smurf1 and inhibiting the degradation of BMPR1A, thus promoting the growth and LN metastasis of NSCLC cells.

To further explore the underlying mechanism through which NADK promoted the metastasis of NSCLC cells, we examined the EMT marker (E-Cadherin and Vimentin) in cells with NADK overexpression. It was found that NADK did not affect the expression of E-Cadherin and Vimentin (Supplementary Fig. 5G), suggesting that NADK promoted the progression of NSCLC independent of EMT. Together, NADK promotes the progression of NSCLC by activating BMP/ID1 pathway (Figure 7).

## Discussion

Metastasis is the leading cause of high mortality in NSCLC. The median survival time for patients with NSCLC is from 14 to 17 months when distant metastasis occurs^30^. This study revealed that the expression of NADK in patients with NSCLC was increased and negatively correlated with survival. NADK promoted the metastasis of NSCLC cells to lymph nodes. These observations suggested that NADK might be a biomarker of lymph node metastasis and a potential therapeutic target for lymph node metastasis in NSCLC.

The most important finding of this study was the discovery of a non-metabolic function of NADK. As the only rate-limiting enzyme in the de novo synthesis of NADPH, NADK mediated the conversion of NAD to NADP^31^. A few studies have investigated the function of NADK in tumors. In pancreatic cancer, PKC phosphorylated NADK, increased its activity, and promoted NADPH synthesis^20^. The increased activity of cytoplasmic NADK led to an increase in NADP level^31^. As a natural PARP inhibitor, NADP has been reported to inhibit DNA repair and promote the apoptosis of cancer cells^32^. However, NADPH, as a reductant, might inhibit tumor progression, eliminate ROS around tumor cells and reshape the tumor microenvironment 33. In addition, NADK mRNA was the target of the miR-690 in the liver, skeletal muscle and adipose tissue and regulated the inflammatory response and insulin signal in macrophages^34^. In recent years, many metabolic enzymes were reported to have non-metabolic functions. Whether NADK had non-metabolic functions was unclear. In this study, we have demonstrated that NADK inhibited the ubiquitination and degradation of BMPR1A through non-metabolic functions.

Currently, multiple potential inhibitors against NADK have been developed. NADK is inhibited by thionicotinamide adenine dinucleotide (NADS) and thionicotinamide adenine dinucleotide phosphate (NADPS). The prodrug thionicotinamide (TN) is intracellularly converted to NADS and NADPS^22^. It acts as a dual inhibitor of NADK and glucose-6-phosphate dehydrogenase (G6PD). Through cooperating with other ROS-inducing chemotherapeutic agents, TN has been reported to delay tumor growth in a mouse model of colon cancer and diffuse large B-cell lymphoma (DLBCL). Since the synthesis of D-2HG by mutant IDH requires NADPH, targeting NADK to inhibit the synthesis of NADPH suggests a novel potential treatment for IDH-mutant tumors 35, 36. Therefore, it is of great importance to study the therapeutic effects of NADK inhibitors in lung cancer.

Another important finding of this study was that ID1 mediated the biological functions of NADK. ID1 has been reported as the downstream effector of the BMPs signalling pathway. Our study found that NADK regulated the transcription of ID1 through the BMPs signalling pathway. Neuron-specific enolase promoted stem cell-like characteristics of small cell lung cancer cells by activating the BMP2/Smad/ID1 pathway^37^. ID1 has also been reported to play an important role in EGFR- and Kras-mutant NSCLC. ID1 promoted the epidermal-mesenchymal transition (EMT), resulting in resistance to the drug osimertinib in NSCLC cells with the EGFR T790M mutation 38. In NSCLC with Kras mutation, ID1inhibited the expression of PD-L1 and increased the tumor-infiltrating CD8+ T cells, thereby inhibiting the tumor growth 39. ID1 has also been shown to promote the proliferation, liver metastasis and colonization of lung cancer cells 40. Recently, many drugs, such as scutellaria flavonoids, fucoidan, berberine, tetramethylpyrazine, crizotinib, cannabidiol and vinblastine, have been shown to exert antitumor functions by inhibiting ID1-related pathways 41-43. Considering previous reports and our present study, the combination of NADK and ID1 inhibitors might be a novel strategy to treat NSCLC.

## Conclusion

Our study reveals that NADK promotes the migration, invasion, growth, LN metastasis and tumorigenesis of NSCLC cells by activating BMPs/ID1 signalling (Figure 7).

## Materials and Methods

### Cell culture and clinical NSCLC samples

Human NSCLC cell lines (A549, H1299, H157 and H520), normal bronchial epithelial cell line (HBE), mouse NSCLC cell line (LLC, Lewis lung carcinoma cells) and HEK293T cells were obtained from the Cell Bank of the Chinese Academy of Sciences. All human NSCLC cell lines were cultured in RPMI 1640 medium, whereas other cells were cultured in DMEM. FBS (10%) and antibiotics (100 U/mL penicillin and 100 mg/mL streptomycin) were added to the media. All cells were cultured in a constant temperature incubator (5% CO_2_, 37℃). Cell transfection was performed using Lipofectamine 8000 according to the instructions.

The following reagents were used for cell experiments: Recombinant BMP-2 protein (Sino Biological, 10426-HNAE) and doxycycline hydrochloride (dox; Sangon, A600889). All cells were freshly thawed before experiments and cells older than 8 weeks were not used in the study. All cell lines were frequently assayed for Mycoplasma using Mycoplasma Stain Assay Kit (Beyotime) to ensure they were free of Mycoplasma contamination. NSCLC tissues and matched adjacent nontumor tissues were collected from Xiangya Hospital, Central South University, with informed consent from the patients. This study was approved by Xiangya Hospital, Central South University.

### Plasmid and Transfection

The coding sequence (CDS) of NADK was inserted into the pLVX-IRES-puro vector. The shRNAs targeting NADK were designed with the help of the Sigma website and cloned into the pLKO.1-puro vector, whereas the shRNAs targeting ID1 cloned into the pGreen vector. The list of shRNA sequences was provided in Table S1.

Lentivirus was packaged in HEK293T cells with psPAX2 and pMD2.G as the packaging plasmids. After being concentrated in PEG8000, the collected virus-containing solution was centrifuged for 1 hour (4℃, 1,600×g). After removing the supernatant, the precipitated virus was dissolved in 2 mL of DMEM. Cells were seeded into a 6-well plate with a density of 30-40%. The next day, 400 mL of the lentiviral suspension was added to the cells and placed in a constant temperature incubator for incubation overnight. Two days later, the cells were cultured with puromycin (2 μg/mL) for 4 days. Then, the resistant cells were pooled, and Western blotting was used to detect the expression of NADK and ID1.

### qPCR

TRIzol (Invitrogen) was used to extract RNA, and 1 μg of RNA was then reversely transcribed into cDNA using a PrimeScript RT Kit (Takara) according to the instructions. A SYBR Green Kit and CFX96 real-time fluorescent qPCR detection system (Bio-Rad) were used for qPCR with Actin as the internal reference. The Ct (Actin-NADK) method was used to calculate the expression of the target gene. The 2^-ΔΔCt^ method was used to calculate the relative expression levels of the target genes. See **Table S1** for the details of the primer sequences used in the experiment.

### Western blotting

The cells were cleaned twice with PBS, and lysed on ice in the RIPA Lysis buffer, which contained a protease inhibitor and a phosphatase inhibitor. The supernatant was collected after the cell lysis was centrifuged (Max speed, 15min). Then the protein concentration was quantified using the BCA protein detection kit. Equal amounts of protein were taken for SDS-PAGE. After separation, the protein was transferred onto the PVDF membrane and incubated with a primary antibody at 4°C overnight. Then, they were incubated with the HRP-conjugated secondary antibody for 1-2h at room temperature. The immune signal was detected with a chemiluminescence reagent (Millipore, WBKLS0050), and analyzed with Image Lab software. The primary antibodies used were as follows: NADK (Proteintech, 15548-1-AP, 1:2000), beta-Tubulin (Proteintech, 10068-1-AP, 1:3000), GAPDH (Proteintech, 10494-1-AP, 1:10000), Actin (Santa Cruz, sc-8432, 1:3000), Flag-tag (DDDDK-Tag) (Proteintech, 20543-1-AP, 1:3000), Myc-Tag (Cell Signaling Technology, 2276S, 1:3000), HA-tag (Proteintech, 66006-Ig,1:3000), Smad1 (Proteintech, 10429-1-AP, 1:3000), Phospho-Smad1(Ser463/465)/Smad5(Ser463/465)/Smad9(Ser465/467) (Cell Signaling Technology, 13820S, 1:1000), ID1 (Proteintech, 67827Ig, 1:2000), Smurf1 (Proteintech, 55175-1-AP, 1:2000) and ubiquitin (Proteintech, 10201-2-AP, 1:1000).

### Immunohistochemistry (IHC)

The tissue arrays were purchased from Shanghai Outdo Biotech Co., Ltd. The clinical features of the tissue array were listed in **Table S2**. After dewaxing and rehydration, the tissue sections were put in the EDTA solution, and antigen recovery was performed at 100°C for 30min. After natural cooling to room temperature, the activity of endogenous peroxidase was blocked with endogenous peroxidase blocking solution. After being washed three times in PBS, the tissue sections were incubated with 3% Normal Goat Serum in PBS for 30min. Then, incubated with the anti-NADK (Proteintech, 15548-1-AP, 1:200), Anti-phospho-SMAD1/5/9 (phospho S463/465/467) (Abcam, ab92698, 1:500), anti-ID1 (Proteintech, 67827-1-Ig, 1:500), anti-Flag-tag (Proteintech, 20543-1-AP; 1:600), anti-Ki67 (ABclonal, WH153065, 1:500) and anti-Ovalbumin (Abcam, ab181688) at 4°C overnight.

The next day, the sections were washed three times in PBS and incubated with the secondary antibody at 37°C for 1h. The immunohistochemical signal was detected with 3,3,0-diaminobenzidine (DAB), and hematoxylin was used for nuclear staining of all the sections. Both the staining intensity and protein expression level were automatically scored by the Inform system. The NADK scores less than 69 were identified as “low expression of NADK”; NADK scores equal to or over than 69 were identified as “high expression of NADK”. The survival curve was drawn using the Kaplan-Meier method, and the log-rank test was used for survival analysis.

### Immuno-precipitation (IP)

To detect the exogenous interaction between NADK and Smurf1, the tagged NADK(Flag-NADK) and Smurf1(HA-Smurf1) plasmids were transfected into HEK293T cells. 36h after transfection, HEK293T cells were lysed with an IP lysis buffer (50mM Tris-HCl, pH 8.0, 150mM NaCl, 1%NP-40, protease and phosphatase inhibitor). The supernatant was collected after centrifugation (12, 000g, 15min) at 4°C. Flag-beads (Sigma, A2220) or HA-beads (Thermo Fisher, 88837) were added to the supernatant for incubation 3h at 4°C. Then, the beads were washed 3 times in TBS buffer. The immunoprecipitated proteins were eluted with 1×loading buffer, and heated at 100°C for 10 min. Then, western blotting analysis was performed.

To further confirm the interaction between the endogenously expressed NADK and Smurf1 in the NSCLC cells, the IP lysis buffer containing protease and phosphatase inhibitor was used for lysis. Equal amounts of protein were taken, and 2μg NADK antibody and 2μg Smurf1 antibody was added for incubation overnight at 4°C, respectively. The next day, 25μL Protein A/G beads (Bimake, B23202) was added for incubation 2h at 4°C. The beads were washed three times with the TBS buffer, then 1×loading buffer was added for heat denaturation, and western blotting analysis was performed.

### Ubiquitination analysis

The tagged NADK(Flag-NADK), Smurf1 (HA-Smurf1) and BMPR1A(Myc-BMPR1A) plasmids were transfected into HEK293T cells. 30h after transfection, HEK293T cells were treated with MG132 at a final concentration of 10μM for 4h, IP lysis buffer (50mM Tris-HCl, pH 8.0, 150mM NaCl, 1%NP-40, protease and phosphatase inhibitor) were used to harvest HEK293T cells. The supernatant was collected after centrifugation (12,000g, 15min). Myc-beads (Thermo Fisher, 88844) were added to the supernatant for incubation of 3h at 4°C. Then, the beads were washed 3 times in TBS buffer, mixed with 1×loading buffer added, and heated at 100°C for 10 min. Then, western blotting analysis was performed.

### Tumorigenesis model and LN metastasis model in C57BL/6J mice

All work related to animals was approved by the Animal Ethics Committee of the Central South University. C57BL/6J mice (Male, 5-6 weeks old) were purchased and housed in barrier facilities on a 12 h light/dark cycle at temperature 18-22 °C and humidity 50-60%. LLC cells were infected with virus expressing chicken protein Ovalbumin which could be probed by IHC staining as the marker for the metastatic tumor cells.

Five C57BL/6J mice were included in each group. 2×10^5^ control cells and LLC cells with overexpression or knockdown of NADK, were injected subcutaneously at each point. The tumor volume was calculated according to the following formula: volume = (length×width^2^)/2. The mice were killed three weeks after the start of the experiment to harvest the tumors. IHC staining was preformed to determine the expression of NADK, phospho-SMAD1/SMAD5/SMAD9 (phospho S463/465/467), ID1, anti-Flag-tag and Ki67.

The LN metastasis model was performed as previously reported^44^. Briefly, mice were randomly divided into two groups (n = 6/group). Each group was further equally divided and injected with control or NADK-overexpressing LLC cells (2 × 10^5^) at the footpad, and another two groups were injected with negative control cells and LLC cell (2 × 10^5^) with knock-down NADK. A month after the inoculation, mice were euthanized. The primary footpad tumors were paraffin-embedded and subjected to hematoxylin-eosin and IHC staining. LNs were first measured to calculate volumes. LNs were used for IHC staining with anti-Ovalbumin antibody to determine the proportion of LN-spread LLC cells.

### Soft agar assay

When the confluence reached 60-70%, the cells were digested, and a cell suspension was prepared. Lower-layer gel (20% FBS, 40% 2×RPMI 1640 (Basal Medium Eagle), 40% 1.25% Agar) was prepared. 400μL of gel was added to each well in the 24-well plate. The gel was placed in an incubator at 37°C. The gel was solidified for later use. Upper-layer gel (25% FBS, 37.5% 2×RPMI 1640, 37.5% 1% Agar, 0.8% 2mM L-glutamine) was prepared and mixed evenly with the cell suspension. 400μl (containing 4×10^3^ cells) was added to each well and placed in a constant temperature incubator (37°C, 5% CO_2_) for 10-14 days. 5 fields of view were selected randomly under the microscope for colony counting.

### CCK8

The cells were seeded into a 96-well plate, with 1×10^3^ cells in each well, and cultured in the incubator (37°C, 5% CO_2_). The next day, the old medium was replaced with a fresh medium containing 10% CCK8, which was placed in the incubator for 2h of incubation to measure its absorbance at 450 nm. Tests were performed on each day until day six.

### Transwell invasion assay and migration assay

70µL of cytokines-free Matrigel diluted in Basic DMEM medium (30:1000) was added to the central of the bottom membrane of the upper chamber, the supernatant was discarded after 30 min. 2×10^4^ cells (1×10^5^ /ml) resuspended in medium containing 0.1% FBS were added to the upper chamber, and 500μl medium containing 30% FBS was put in the lower well. After Cultured in the incubator (37°C, 5% CO_2_) for 48h, wells were washed three times with the PBS buffer, then fixed with 4% paraformaldehyde and stained with crystal violet. Migration assay was preformed almost the same as the invasion assay except for matrigel coat, and cultured for 24h. Five fields of view were randomly selected for observation by a 20‐fold microscope, then photographed and counted with Image J software to calculate the relative invasion rate.

### Wound-healing assay

When cells reached the 100% confluence, a straight line was scratched with a 200µL pipette tip on the cell monolayer. The wounds were observed and images were taken at 20× magnification at 0, 24 and 48h or 0, 12 and 24h. Images were analysed using the ImageJ software.

### Statistical analysis

Statistical analyses are performed using the SPSS version 23.0 statistical software package and Graph-Pad Prism 8 version 8.0.2 software (GraphPad software, La Jolla, CA, USA). Statistical tests for data analysis included log-rank test, χ2 test (two-sided), and Student's t test (two-sided). P < 0.05 was considered statistically significant.

## Supplementary Material

Supplementary figures and tables.

## Figures and Tables

**Table 1:** The correlation between the NADK expression and the clinical features of lung cancer

Characteristic	Total	NADK Expression		χ2	p-Value
		Low n=48	High n=49		
**Gender**					
Male	55	28	27	0.748	0.838
Female	42	20	22		
**Age**					
>60	47	21	26	1.242	0.312
≤60	50	28	22		
**Pathological grade**					
Ⅰ,Ⅰ-Ⅱ	9	6	3	3.539	0.170
Ⅱ,	54	30	24		
Ⅱ-Ⅲ,Ⅲ	34	13	21		
**Tumor size**					
≥30	46	21	25	0.514	0.544
<30	51	27	24		
**Lymph node metastasis**					
Yes	53	17	36	15.894	**0.001**
No	44	32	12		
**TNM stage**					
Ⅰ	32	23	9	8.725	**0.013**
Ⅱ, Ⅱ-Ⅲ	33	13	20		
Ⅲ,Ⅳ	32	13	19		
**AJCC Cancer Stage, 7^th^ Edition**					
Ⅰ	30	21	9	7.095	**0.029**
Ⅱ, Ⅱ-Ⅲ	35	15	20		
Ⅲ,Ⅳ	31	12	19		

## Abbreviations

NADK: Nicotinamide adenine dinucleotide kinase
NSCLC: Non-small cell lung cancer
LN: Lymph node
LNM: Lymph node metastasis
co-Smad: co-mediator Smad
ROS: Reactive oxygen species
MEs: Malic enzymes
TN: Thionicotinamide
IHC: Immunohistochemistry
DEGs: Differentially expressed genes
NADS: Thionicotinamide adenine dinucleotide
NADPS: Thionicotinamide adenine dinucleotide phosphate
G6PD: Glucose-6-phosphate dehydrogenase
DLBCL: Diffuse large B-cell lymphoma
EMT: Epidermal-mesenchymal transition

## References

[1] Siegel R (2023). Cancer statistics, 2023. CA: a cancer journal for clinicians.

[2] Clark SB, Alsubait S (2022). Non Small Cell Lung Cancer. Treasure Island (FL): StatPearls.

[3] Herbst R (2018). The biology and management of non-small cell lung cancer. Nature.

[4] du Bois H (2021). Tumor-draining lymph nodes: At the crossroads of metastasis and immunity. Science immunology.

[5] Karaman S, Detmar M (2014). Mechanisms of lymphatic metastasis. The Journal of clinical investigation.

[6] Diao X (2022). Lymphatic metastasis in non-small cell lung cancer: recent discoveries and novel therapeutic targets. Cancer communications (London, England).

[7] Wang C (2020). Clinicopathological variables influencing overall survival, recurrence and post-recurrence survival in resected stage I non-small-cell lung cancer. BMC Cancer.

[8] Bille A (2017). Incidence of occult pN2 disease following resection and mediastinal lymph node dissection in clinical stage I lung cancer patients. Eur J Cardiothorac Surg.

[9] Huang F (2020). BMP2 signalling activation enhances bone metastases of non-small cell lung cancer. J Cell Mol Med.

[10] Mihajlović J (2019). Inhibition of bone morphogenetic protein signaling reduces viability, growth and migratory potential of non-small cell lung carcinoma cells. J Cancer Res Clin Oncol.

[11] Jiang B (2022). Lysosomal protein transmembrane 5 promotes lung-specific metastasis by regulating BMPR1A lysosomal degradation. Nature communications.

[12] Liu Y (2018). Knockdown of Bone Morphogenetic Proteins Type 1a Receptor (BMPR1a) in Breast Cancer Cells Protects Bone from Breast Cancer-Induced Osteolysis by Suppressing RANKL Expression. Cell Physiol Biochem.

[13] Lee HW (2022). Chun, BMPR1A Promotes ID2-ZEB1 Interaction to Suppress Excessive Endothelial to Mesenchymal Transition. Cardiovasc Res.

[14] Hover LD (2015). Small molecule inhibitor of the bone morphogenetic protein pathway DMH1 reduces ovarian cancer cell growth. Cancer Lett.

[15] Fukuda T (2020). Miyazono, C.H. Heldin, BMP signaling is a therapeutic target in ovarian cancer. Cell Death Discov.

[16] Ma J (2021). Inhibiting Endothelial Cell Function in Normal and Tumor Angiogenesis Using BMP Type I Receptor Macrocyclic Kinase Inhibitors. Cancers.

[17] Prasad S (2017). Reactive oxygen species (ROS) and cancer: Role of antioxidative nutraceuticals. Cancer Lett.

[18] Trachootham D (2009). Targeting cancer cells by ROS-mediated mechanisms: a radical therapeutic approach?. Nat Rev Drug Discov.

[19] Hoxhaj G (2019). Direct stimulation of NADP(+) synthesis through Akt-mediated phosphorylation of NAD kinase. Science.

[20] Schild T (2021). NADK is activated by oncogenic signaling to sustain pancreatic ductal adenocarcinoma. Cell Rep.

[21] Tsang YH (2016). Functional annotation of rare gene aberration drivers of pancreatic cancer. Nat Commun.

[22] Tedeschi PM (2016). NAD+ Kinase as a Therapeutic Target in Cancer. Clin Cancer Res.

[23] Wang J (2022). A non-metabolic function of hexokinase 2 in small cell lung cancer: promotes cancer cell stemness by increasing USP11-mediated CD133 stability. Cancer communications (London, England).

[24] Owens P (2015). Inhibition of BMP signaling suppresses metastasis in mammary cancer. Oncogene.

[25] Wu CK (2022). BMP2 promotes lung adenocarcinoma metastasis through BMP receptor 2-mediated SMAD1/5 activation. Sci Rep.

[26] Langenfeld EM (2005). Expression of bone morphogenetic proteins in human lung carcinomas. Ann Thorac Surg.

[27] Jia Y (2016). Tetramethylpyrazine inhibits tumor growth of lung cancer through disrupting angiogenesis via BMP/Smad/Id-1 signaling. Int J Oncol.

[28] Langenfeld E (2013). Small molecule antagonist of the bone morphogenetic protein type I receptors suppresses growth and expression of Id1 and Id3 in lung cancer cells expressing Oct4 or nestin. Mol Cancer.

[29] Pillai S (2011). ID1 facilitates the growth and metastasis of non-small cell lung cancer in response to nicotinic acetylcholine receptor and epidermal growth factor receptor signaling. Mol Cell Biol.

[30] Simeone JC (2019). Treatment patterns and overall survival in metastatic non-small-cell lung cancer in a real-world, US setting. Future Oncol.

[31] Rather GM (2021). Tedeschi, In cancer, all roads lead to NADPH. Pharmacol Ther.

[32] Bian C (2019). NADP(+) is an endogenous PARP inhibitor in DNA damage response and tumor suppression. Nat Commun.

[33] Ju HQ (2020). NADPH homeostasis in cancer: functions, mechanisms and therapeutic implications. Signal Transduct Target Ther.

[34] Ying W (2021). MiR-690, an exosomal-derived miRNA from M2-polarized macrophages, improves insulin sensitivity in obese mice. Cell Metab.

[35] Tateishi K (2015). Cahill, Extreme Vulnerability of IDH1 Mutant Cancers to NAD+ Depletion. Cancer Cell.

[36] Pramono AA (2020). NAD- and NADPH-Contributing Enzymes as Therapeutic Targets in Cancer: An Overview. Biomolecules.

[37] Lu L (2022). Neuron-specific enolase promotes stem cell-like characteristics of small-cell lung cancer by downregulating NBL1 and activating the BMP2/Smad/ID1 pathway. Oncogenesis.

[38] Liu K (2021). ID1 mediates resistance to osimertinib in EGFR T790M-positive non-small cell lung cancer through epithelial-mesenchymal transition. BMC Pulm Med.

[39] Baraibar I (2020). Gil-Bazo, Id1 and PD-1 Combined Blockade Impairs Tumor Growth and Survival of KRAS-mutant Lung Cancer by Stimulating PD-L1 Expression and Tumor Infiltrating CD8(+) T Cells. Cancers (Basel).

[40] Castañón E (2017). The inhibitor of differentiation-1 (Id1) enables lung cancer liver colonization through activation of an EMT program in tumor cells and establishment of the pre-metastatic niche. Cancer Lett.

[41] Zhao Z (2019). Scutellaria Flavonoids Effectively Inhibit the Malignant Phenotypes of Non-small Cell Lung Cancer in an Id1-dependent Manner. Int J Biol Sci.

[42] Zhao Z (2019). Baicalein Inhibits Orthotopic Human Non-Small Cell Lung Cancer Xenografts via Src/Id1 Pathway. Evid Based Complement Alternat Med.

[43] Zhao Z (2020). Inhibitor of Differentiation 1 (Id1) in Cancer and Cancer Therapy. Int J Med Sci.

[44] Liu L (2015). TBL1XR1 promotes lymphangiogenesis and lymphatic metastasis in esophageal squamous cell carcinoma. Gut.

