# Supplementary material for: Nicotinamide adenine dinucleotide kinase promotes lymph node metastasis of NSCLC via activating ID1 expression through BMP pathway
Source: Int J Biol Sci. 2023 Jun 19;19(10):3184–99. doi: 10.7150/ijbs.84322 (PMC10321276; doi:10.7150/ijbs.84322)
Supplement: Supplementary file 1 — Supplementary figures and tables. [file ijbsv19p3184s1.pdf]

Supplementary Materials

The supplementary materials included 6 figures and 3 tables.

1. Supplementary figures

Supplementary Figure 1. The expression of NADK is elevated in lung cancer.

Figure S1A. Relative mRNA levels of NADK in human NSCLC cell (H157, H460, A549, H520 and H1299) and human bronchial epithelial cell (HBE) were analysed with a *t* test. **S1B.** The mRNA levels of NADK in 56 NSCLC tissues and paired adjacent tissues were analysed with a paired *t* test. **S1C and 1D.** Representative IHC images showing the expression of NADK in 3 NSCLC tissues and matched adjacent nontumor tissues. The protein levels of NADK in the NSCLC tissues were scored. The tissue array contained 81 NSCLC tissues and paired adjacent tissues (**S1D**). Paired *t* test was performed. **S1E.** Western blot was used to measure the levels of NADK protein in 9 NSCLC tissues and paired adjacent tissues. N, the adjacent nontumor tissue; T, the tumor tissue. HSP90 was used as the internal reference. **S1F.** The GEPIA database was used to analyse the correlation between the expression of NADK and the progression-free survival (PFS) of NSCLC patients (**Left**), Overall Survival (OS) (**Right**) of patients with LUSC. The scale bars were indicated. **, P<0.01; ****, P<0.0001.

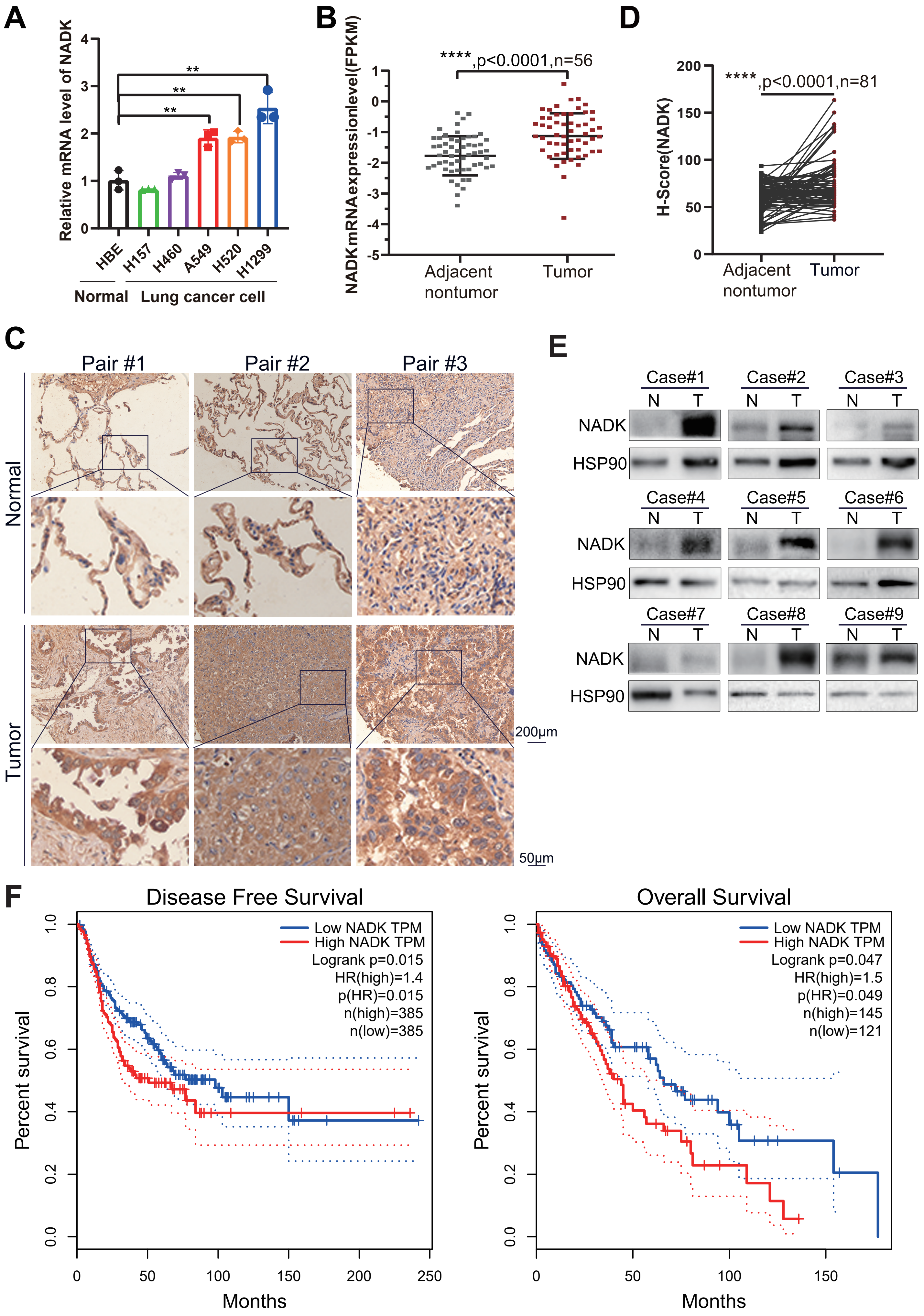

A
Relative mRNA level of NADK
HBE
H157
H460
A549
H520
H1299
Normal
Lung cancer cell
**
B
NADK mRNA expression level(FPKM)
****,p<0.0001,n=56
Adjacent nontumor
Tumor
D
H-Score(NADK)
****,p<0.0001,n=81
Adjacent nontumor
Tumor
C
Pair #1
Pair #2
Pair #3
Normal
Tumor
200μm
50μm
E
Case#1
Case#2
Case#3
Case#4
Case#5
Case#6
Case#7
Case#8
Case#9
N
T
NADK
HSP90
F
Disease Free Survival
Low NADK TPM
High NADK TPM
Logrank p=0.015
HR(high)=1.4
p(HR)=0.015
n(high)=385
n(low)=385
Percent survival
Months
Overall Survival
Low NADK TPM
High NADK TPM
Logrank p=0.047
HR(high)=1.5
p(HR)=0.049
n(high)=145
n(low)=121
Percent survival
Months

Supplementary Figure 2. NADK enhances proliferation and anchorage-independent growth of NSCLC cells.

Figure **S2A**. A CCK8 assay was performed to detect the effects of NADK knockdown on the proliferation of A549 and H157 cells. **S2B**. The effects of overexpressed NADK on the proliferation of NSCLC cells (A549, H1299, HBE) were detect with the CCK8 assay. **S2C-D.** Soft-agar assay was used to measure the effects of NADK knockdown on the anchor-independent growth of A549, H157 and H520 cells. **S2E-F.** Soft-agar assay was performed to examine the effect of NADK overexpression on the colony formation of A549, H1299 and HBE cells (**S2E**). The colonies were quantified and analysed (**S2F**). The scale bars were indicated. *, $P<0.05$; **, $P<0.01$; ***, $P<0.001$; ****, $P<0.0001$.

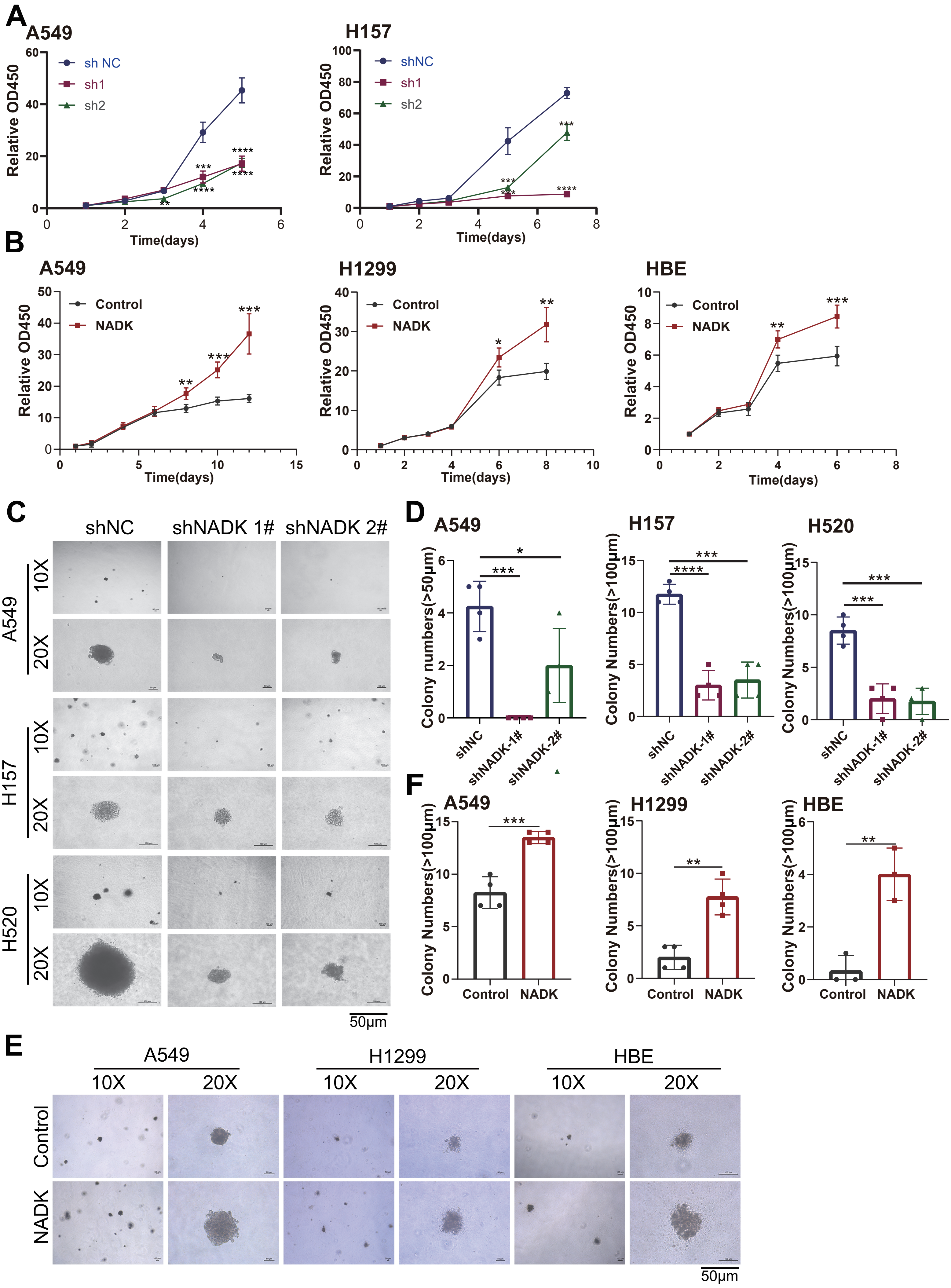

A
A549
Relative OD450
sh NC
sh1
sh2
Time(days)
H157
shNC
sh1
sh2
Relative OD450
Time(days)
B
A549
Control
NADK
Relative OD450
Time(days)
H1299
Control
NADK
Relative OD450
Time(days)
HBE
Control
NADK
Relative OD450
Time(days)
C
shNC
shNADK 1#
shNADK 2#
A549
H157
H520
10X
20X
50μm
D
A549
Colony numbers(>50μm)
shNC
shNADK-1#
shNADK-2#
H157
Colony Numbers(>100μm)
H520
Colony Numbers(>100μm)
F
A549
Colony Numbers(>100μm)
Control
NADK
H1299
HBE
E
A549
H1299
HBE
10X
20X
Control
NADK
50μm

Supplementary Figure 3. NADK promotes the tumorigenicity of NSCLC cells.

Figure **S3A.** The morphology of the xenografts formed by the LLC control cells or LLC cells with the overexpression of NADK. See "materials and methods" for details. **S3B.** Growth curve of xenografts shown in **S3A**. Tumor volumes were measured at indicate time points. (n=5, Error bars represent SEM, *, $P<0.05$; **, $P<0.01$. Unpaired *t* test.) **S3C.** The weight of the xenografts in **S3A** was examined at the end of the experiment. **S3D -S3E.** Representative IHC images of Ki67 for xenografts **shown in S3A** (**S3D**), the expression of Ki67 were scored and analysed (**S3E**). **S3F.** The morphology of the xenografts formed by the LLC cells infected with the NADK shRNA virus or the negative control shRNA virus. **S3G.** Growth curve of xenografts shown in **S3F**. Tumor volumes were measured at indicate time points. (n=5, Error bars represent SEM, *, $P<0.05$; **, $P<0.01$. Unpaired *t* test). **S3H.** The weight of the xenografts shown in **S3F** was measured and analysed with unpaired *t* test. **S3I-S3J.** The expression of Ki67 in LLC-shNadk xenografts was examined by IHC, scored and analysed. The scale bars were indicated. *, $P<0.05$; **, $P<0.01$; ***, $P<0.001$; ****, $P<0.0001$.

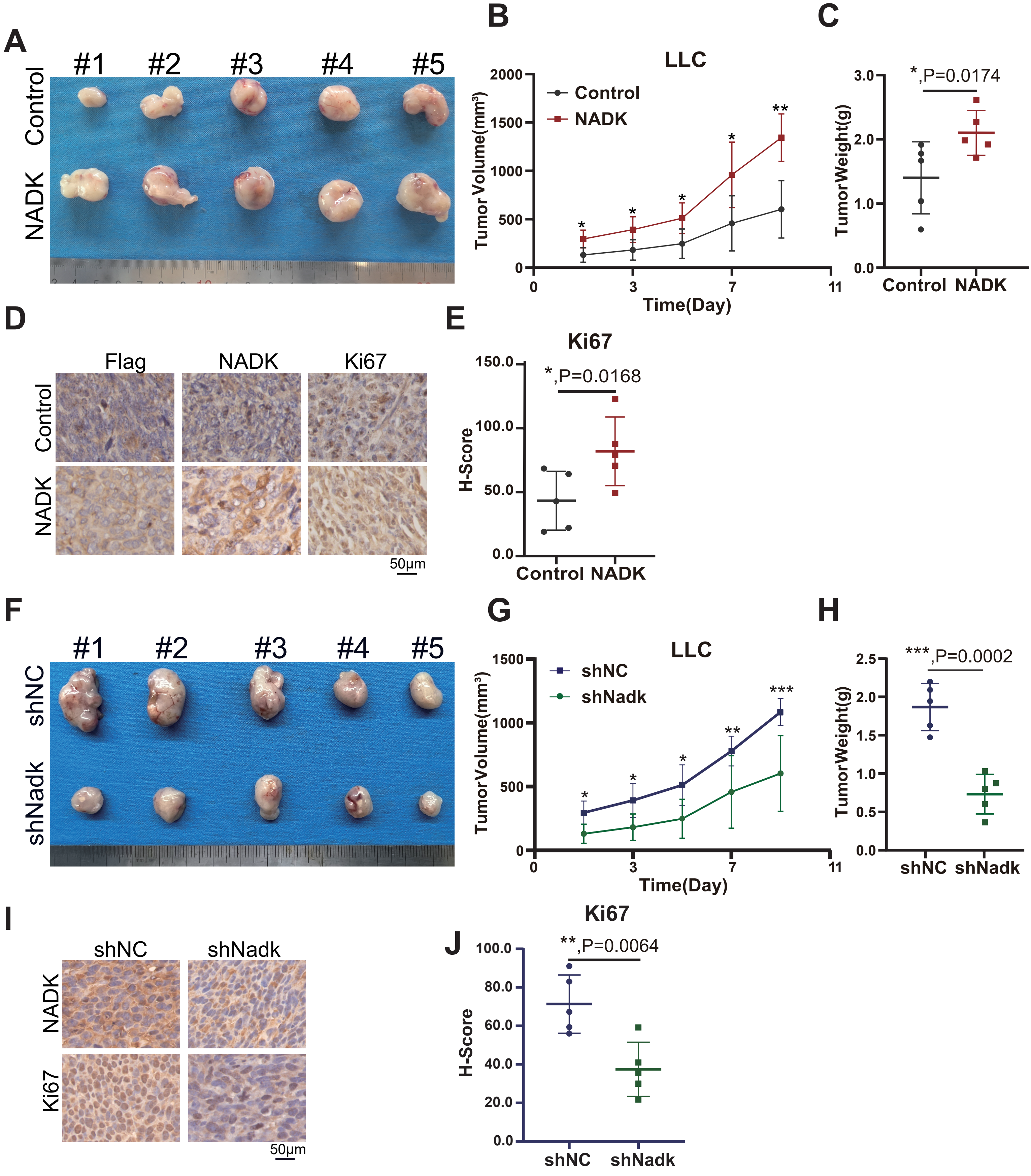
A
#1
#2
#3
#4
#5
Control
NADK
B
LLC
Control
NADK
Tumor Volume(mm³)
2000
1500
1000
500
0
0
3
7
11
Time(Day)
*
*
*
*
**
C
*,P=0.0174
TumorWeight(g)
3.0
2.0
1.0
0.0
Control
NADK
D
Flag
NADK
Ki67
Control
NADK
50μm
E
Ki67
*,P=0.0168
H-Score
150.0
100.0
50.0
0.0
Control
NADK
F
#1
#2
#3
#4
#5
shNC
shNadk
G
LLC
shNC
shNadk
TumorVolume(mm³)
1500
1000
500
0
0
3
7
11
Time(Day)
*
*
*
**
***
H
***,P=0.0002
TumorWeight(g)
2.5
2.0
1.5
1.0
0.5
0.0
shNC
shNadk
I
shNC
shNadk
NADK
Ki67
50μm
J
Ki67
**,P=0.0064
H-Score
100.0
80.0
60.0
40.0
20.0
0.0
shNC
shNadk

Supplementary Figure 4. NADK activates TGFβ signalling.

Figure **S4A.** KEGG pathview analysis shows the enrichment of TGFβ signalling in A549 cells with NADK overexpression. **S4B.** Co-IP was performed to examine the interaction between NADK and key components of TGFβ signalling.

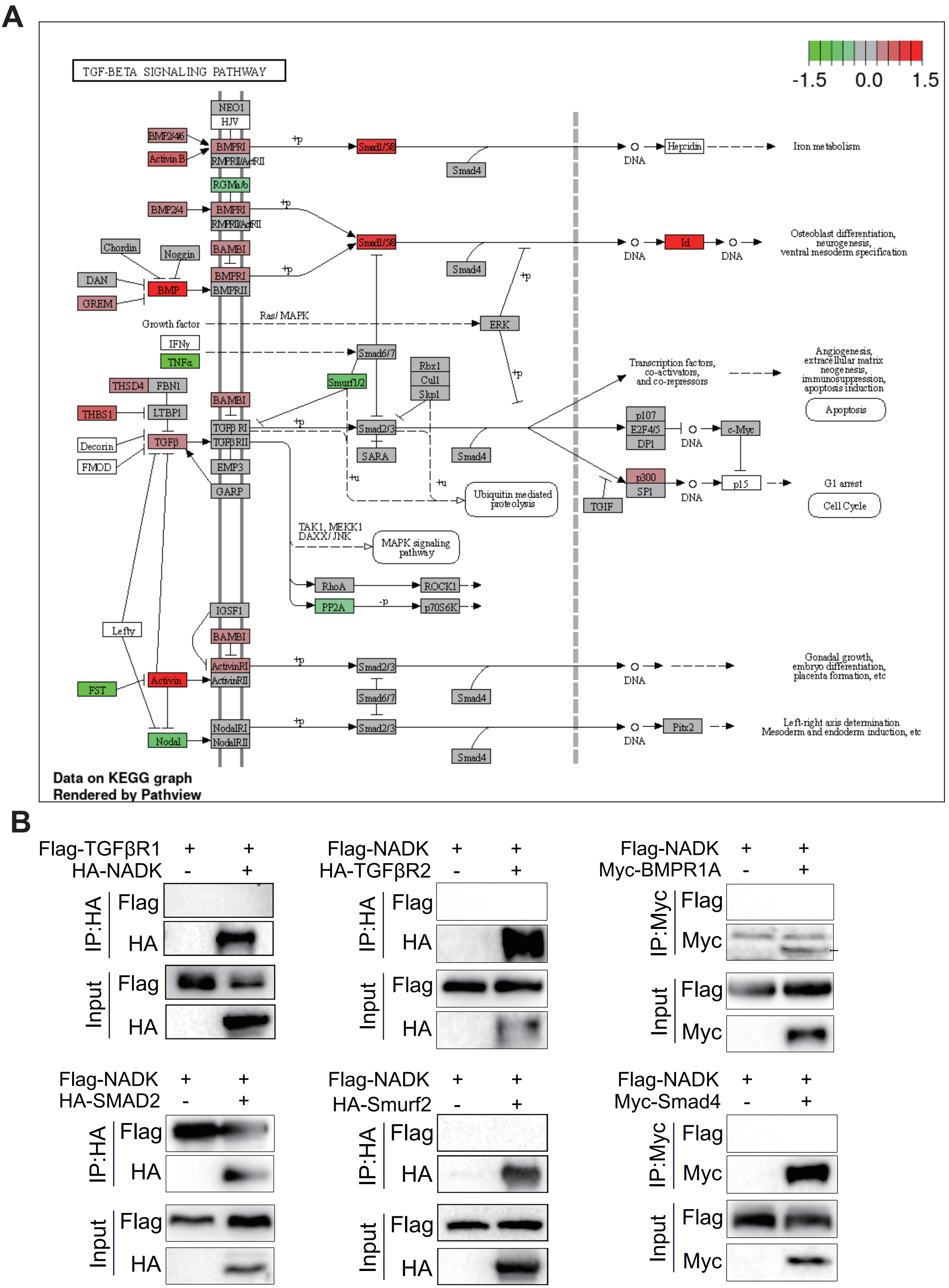

A
TGF-BETA SIGNALING PATHWAY
-1.5
0.0
1.5
NEO1
HJV
BMP2/4/6
Activin B
BMPRI
RMPRII/ActRII
+p
Smad1/5/8
Smad4
DNA
Hepcidin
Iron metabolism
RGMa/b
BMP2/4
Chordin
Noggin
BAMBI
DAN
BMP
BMPRII
GREM
Id
Osteoblast differentiation,
neurogenesis,
ventral mesoderm specification
Growth factor
Ras/ MAPK
ERK
IFNγ
TNFα
Smad6/7
Smurf1/2
Rbx1
Cul1
Skp1
THSD4
FBN1
THBS1
LTBP1
TGFβ RI
TGFβ RII
Decorin
TGFβ
FMOD
EMP3
GARP
Smad2/3
SARA
Transcription factors,
co-activators,
and co-repressors
Angiogenesis,
extracellular matrix
neogenesis,
immunosuppression,
apoptosis induction
Apoptosis
p107
E2F4/5
DP1
c-Myc
p300
SP1
TGIF
p15
G1 arrest
Cell Cycle
Ubiquitin mediated
proteolysis
+u
TAK1, MEKK1
DAXX/ JNK
MAPK signaling
pathway
RhoA
ROCK1
PP2A
-p
p70S6K
IGSF1
Lefty
ActivinRI
ActivinRII
FST
Activin
Gonadal growth,
embryo differentiation,
placenta formation, etc
Nodal
NodalRI
NodalRII
Pitx2
Left-right axis determination
Mesoderm and endoderm induction, etc
Data on KEGG graph
Rendered by Pathview
B
Flag-TGFβR1 + +
HA-NADK - +
IP:HA
Flag
HA
Input
Flag
HA
Flag-NADK + +
HA-TGFβR2 - +
Flag-NADK + +
Myc-BMPR1A - +
IP:Myc
Myc
Flag-NADK + +
HA-SMAD2 - +
Flag-NADK + +
HA-Smurf2 - +
Flag-NADK + +
Myc-Smad4 - +

Supplementary Figure 5. NADK activates BMPs/ID1 signaling axis.

Figure **S5A.** Western blotting was used to detect the level of phosphorylated Smad2 in NADK overexpressed HBE and H1299 cells treated with TGFβ1. **S5B.** Western blotting was performed to measure phosphorylation level of Smad1/5/9 after the treatment of BMP-2 in NADK overexpressed KP and H1299 cells**. S5C** Correlation analysis between ID1 mRNA level and NADK mRNA level in NSCLC sample after mining the TCGA and GTEx databases. **S5D**. Knockdown of ID1 in NADK overexpressed cell lines. **S5E.** Wound healing assay was used to detect the effect of ID1 knockdown on the migration promoted by NADK. Relative migration areas were measured and analysed. **S5F** IHC staining was performed to examine the levels of ID1 protein in xenografts formed by LLC cells overexpressed NADK and LLC control cells. The levels of ID1 protein were quantified and analysed. **S5G**. Western blot was performed to measure the level of EMT marker (E-Cadherin and Vimentin) in cells overexpressing NADK (A547 and H157). The scale bars were indicated. **, $P<0.01$; ***, $P<0.001$.

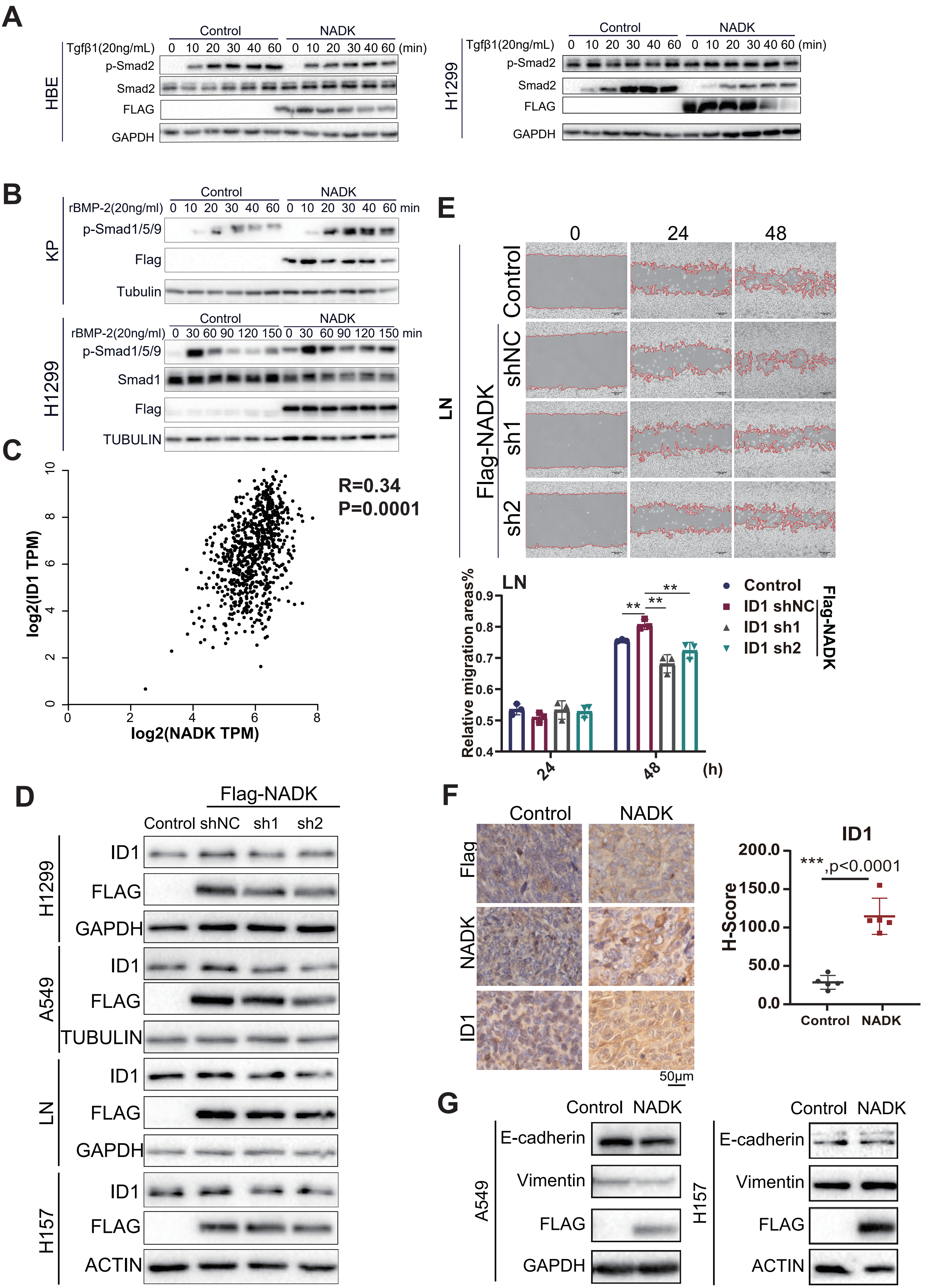

A
Control
NADK
Tgfβ1(20ng/mL) 0 10 20 30 40 60 0 10 20 30 40 60 (min)
p-Smad2
Smad2
FLAG
GAPDH
HBE
H1299
B
rBMP-2(20ng/ml) 0 10 20 30 40 60 0 10 20 30 40 60 min
p-Smad1/5/9
Flag
Tubulin
KP
rBMP-2(20ng/ml) 0 30 60 90 120 150 0 30 60 90 120 150 min
Smad1
TUBULIN
C
R=0.34
P=0.0001
log2(ID1 TPM)
log2(NADK TPM)
D
Flag-NADK
Control shNC sh1 sh2
ID1
FLAG
GAPDH
A549
TUBULIN
LN
H157
ACTIN
E
0 24 48
Control
shNC
sh1
sh2
Flag-NADK
Relative migration areas%
Control
ID1 shNC
ID1 sh1
ID1 sh2
(h)
F
Control NADK
Flag
NADK
ID1
50μm
H-Score
***,p<0.0001
G
E-cadherin
Vimentin

Supplementary Figure 6. Expression of NADK, Ki67, p-Smad1/5/9 and ID1 in the xenografts were examined using IHC.

Figure **S6A.** Representative IHC images of Flag, NADK, Ki67, p-Smad1/5/9 and ID1 performed on xenografts formed by cells overexpressing of NADK. **S6B.** Representative IHC images of NADK, Ki67, p-Smad1/5/9 and ID1 performed on xenografts formed by the cells with NADK knockdown.

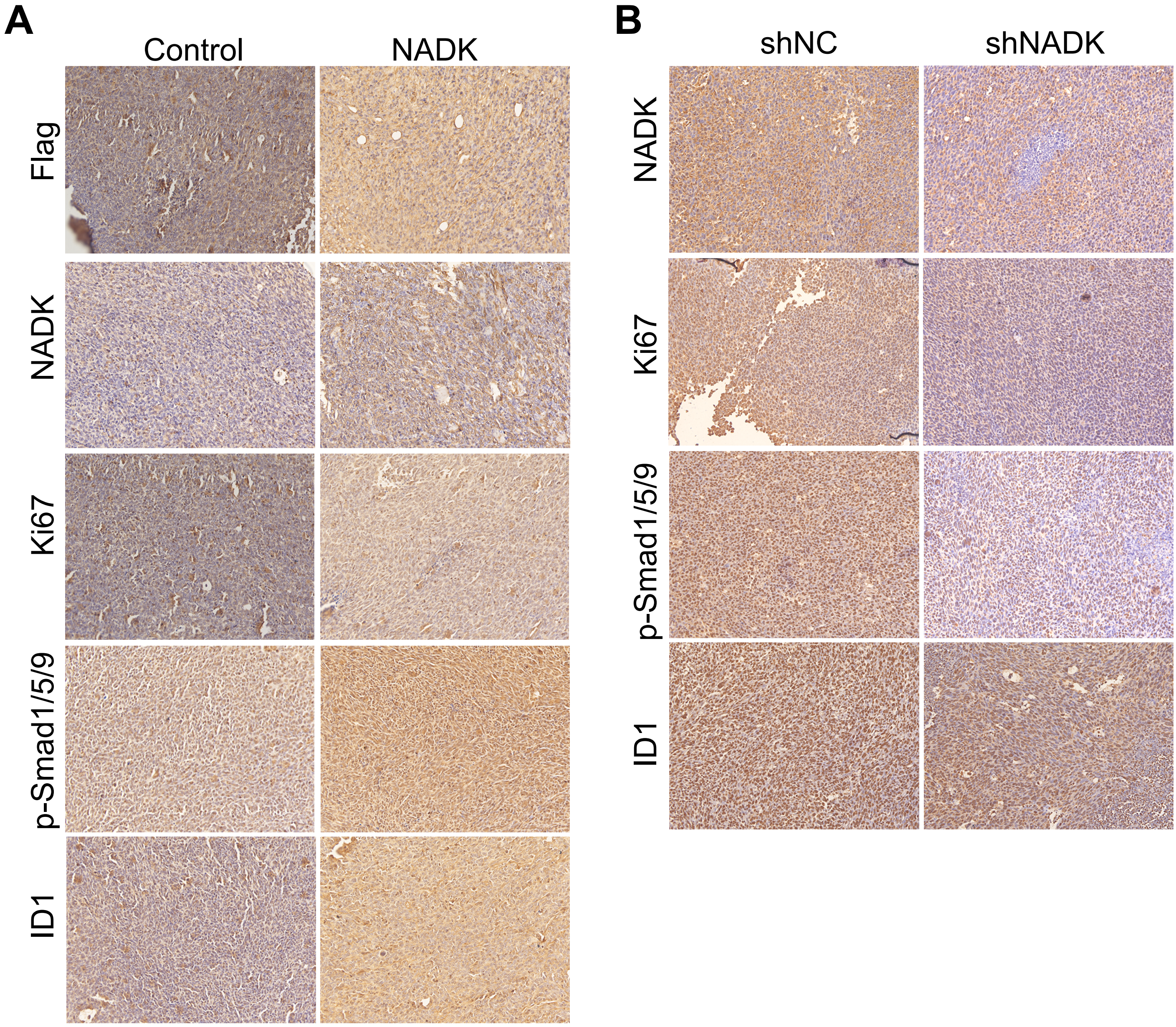

A
Control
NADK
Flag
NADK
Ki67
p-Smad1/5/9
ID1
B
shNC
shNADK
NADK
Ki67
p-Smad1/5/9
ID1

2. Supplemental Table

Table S1. Primers and oligo sequences.

Table S2. NSCLC tissue array and detail pathological information.

Table S3. KEGG Enrichment: A549 overexpressing NADK VS A549 Control cells.

Supplementary Table 1 qPCR, shRNA and over-expression plasmid Primers Sequence

Primer	Forward sequence (5’-3’)	Reverse sequence (5’-3’)
NADK (Homo)-qPCR	ACCTGAAGCAAGGAACACAGC	AGCGGGTAGCATGAGGTAGT
ID1 (Homo)-qPCR	CTGCTCTACGACATGAACGG	GAAGGTCCCTGATGTAGTCGAT
Id1 (Mouse)-qPCR	CCTAGCTGTTCGCTGAAGGC	CTCCGACAGACCAAGTACCAC
Actin (Homo)-qPCR	GAATCAATGCAAGTTCGGTTCC	TCATCTCCGCTATTAGCTCCG
Gapdh (Mouse)-qPCR	AGGTCGGTGTGAACGGATTTG	TGTAGACCATGTAGTTGAGGTCA
NADK shRNA 1#	CCGGGCATTGGAACGTCCGGAAGAACTCGAGTTCTTCCGGACGTTCCAATGCTTTTTG	AATTCAAAAAGCATTGGAACGTCCGGAAGAACTCGAGTTCTTCCGGACGTTCCAATGC
NADK shRNA 2#	CCGGGCATCAGCATCACTACCTCATCTCGAGATGAGGTAGTGATGCTGATGCTTTTTG	AATTCAAAAAGCATCAGCATCACTACCTCATCTCGAGATGAGGTAGTGATGCTGATGC
Nadk shRNA (Mouse)	CACCGTTTCATCACTCACTATAGCA	AAACTGCTATAGTGAGTGATGAAAC
ID1 shRNA 1#	GATCCGACTCGGAATCCGAAGTTGGAATTCAAGAGATTCCAACTTCGGATTCCGAGTTTTTTTG	AATTCAAAAAAACTCGGAATCCGAAGTTGGAATCTCTTGAATTCCAACTTCGGATTCCGAGTCG
ID1 shRNA 2#	GATCCGGCAGGTAAACGTGCTGCTCTATTCAAGAGATAGAGCAGCACGTTTACCTGCTTTTTTG	AATTCAAAAAAGCAGGTAAACGTGCTGCTCTATCTCTTGAATAGAGCAGCACGTTTACCTGCCG
Id1 shRNA 1#(Mouse)	GATCCGGCATGTGTTCCAGCCGACGATTTCAAGAGAATCGTCGGCTGGAACACATGCTTTTTTG	AATTCAAAAAAGCATGTGTTCCAGCCGACGATTCTCTTGAAATCGTCGGCTGGAACACATGCCG
Id1 shRNA 2#(Mouse)	GATCCGGCGAGGTGGTACTTGGTCTGTTTCAAGAGAACAGACCAAGTACCACCTCGCTTTTTTG	AATTCAAAAAAGCGAGGTGGTACTTGGTCTGTTCTCTTGAAACAGACCAAGTACCACCTCGCCG
FLAG-NADK	AAGCTGGGCGGTGGTGGATCCATGGAGATGGAGCAGGAGA	TGCACCGGTACTAGTTCTAGATCAGCCCTCCTCCTCCTCCT
FLAG-NADK TD1	AAGCTGGGCGGTGGTGGATCCATGGAGATGGAGCAGGAGA	TGCACCGGTACTAGTTCTAGACTACAGCACGCTCTTAGGGG
FLAG-NADK TD2	AAGCTGGGCGGTGGTGGATCCGTCATCAAGAAGATGAGGGAC	TGCACCGGTACTAGTTCTAGACTAGTTGCCCTCGATGACCTG
FLAG-NADK TD3	AAGCTGGGCGGTGGTGGATCCGCCGCCGTGGTGCTGAGAAG	TGCACCGGTACTAGTTCTAGATCAGCCCTCCTCCTCCTCCT

Supplementary Table 2 NSCLC tissues array and detail pathological information

Number	H-score	Gender	Age	Totol LM	Posotive LN	LNM Rate	T	N	M	TNM stage	AJCC stage, 7th edition	Pathological grade	Tumor size (cm^3)	Survival time (Month)	Survival status
**E05A0031**	150.2	Male	47	10	10	1	T2a	N1	M0	2b	2A	II	42.875		Lost
**E05A0032**	90.8	Female	72	3	0	0	T1b	N0	M0	1a2	1A	II	9	78	Death
**E05A0036**	135.2	Female	59	15	14	0.933333	T3	N1	M0	3a	3A	II	45.6	38	Death
**E05A0046**	130	Female	66	12	9	0.75	T2a	Nx	M0	2-3	2-3	II	36	49	Death
**E05A0059**	87.2	Male	60	3	0	0	T2b	N0	M0	2a	2A	III	72		Lost
**E05A0060**	41.6	Male	49	1	0	0	T1b	N0	M0	1a2	1A	II	18	116	Alive
**E05A0064**	45.2	Male	66	10	0	0	T3	N0	M0	2B	2B	I-II	180		lost
**E05A0081**	67.8	Female	53	5	0	0	T1b	N0	M0	1a2	1A	II	18	113	Alive
**E05A0104**	132.2	Male	74	9	1	0.111111	T2a	Nx	M0	2-3	2-3	II	8.37333333	33	Death
**E05A0123**	95.2	Male	74	2	0	0	T2a	N0	M0	1a1	1B	II	14	21	Death
**E05A0131**	60	Female	20	8	0	0	T1b	N0	M0	1a2	1A	II-III	12		lost
**E05A0142**	95.8	Male	58	11	0	0	T2a	N0	M0	1b	1B	II-III	24	39	Death
**E05A0146**	95.2	Male	30	10	0	0	T1b	N0	M0	1a2	1A	II-III	18	34	Death
**E05A0149**	163.4	Male	67	20	14	0.7	T2b	Nx	M0	2-3	2-3	III	180	39	Death
**E05A0172**	95.2	Male	57	12	0	0	T2a	N0	M0	1b	1B	II-III	55.125		lost
**E05A0175**	137.6667	Female	25	7	1	0.142857	——	Nx	M0	2-3	2-3	III	140	103	Alive
**E05A0182**	92.6	Female	64	8	0	0	T2a	N0	M0	1b	1B	II	24	15	Death
**E05A0195**	72.8	Female	50	5	4	0.8	T3	N1	M0	3a	3A	I	15	55	Death
**E05A0197**	69	Female	46	13	3	0.230769	T3	N1	M0	3a	3A	III	346.5	10	Death

Supplementary Table 2 NSCLC tissues array and detail pathological information

Number	H-score	Gender	Age	Totol LM	Posotive LN	LNM Rate	T	N	M	TNM stage	AJCC stage, 7th edition	Pathological grade	Tumor size (cm^3)	Survival time (Month)	Survival status
**E05A0201**	68	Male	60	10	0	0	T2a	N0	M0	1b	1B	II	49	62	Death
**E05A0208**	60.6	Male	47	7	1	0.142857	T3	N1	M0	3a	3A	III	180	33	Death
**E05A0209**	70.5	Male	65	18	15	0.833333	T3	N2	M0	3b	3A	III	204	14	Death
**E05A0210**	60.6	Female	58	12	0	0	T2a	N0	M1b	4a	2-3	II-III	63	49	Death
**E05A0211**	70.5	Female	67	5	1	0.2	T2a	N1	M0	2b	2A	II	26.25	13	Death
**E05A0222**	64.4	Male	57	11	0	0	T1b	N0	M0	1a2	1A	II	9.42		lost
**E05A0226**	63.6	Female	55	15	0	0	T1b	N0	M0	1a2	1A	I	18.75		lost
**E05A0238**	84.2	Male	51	15	2	2	——	Nx	M0	2-3	2-3	II	18		lost
**E05A0252**	64.8	Female	50	4	0	0	T1b	N0	M0	1a2	1A	II	22.5	92	Alive
**E05A0255**	68.6	Female	68	8	0	0	T1b	N0	M0	1a2	1A	II	7.2	91	Alive
**E05A0270**	63.6	Male	55	8	0	0	T2a	N0	M0	1b	1B	II-III	36	91	Alive
**E05A0272**	59.6	Female	76	1	0	0	T1b	N0	M0	1a2	1A	II-III	12	15	Death
**E05A0273**	36.6	Female	56	6	0	0	T1	N0	M0	1a2	1A	II	1.04666667		lost
**E05A0274**	50.4	Female	62	25	11	0.44	T3	N3	M0	3c	3B	II-III	10.5	9	Death
**E05A0277**	60.6	Male	74	5	2	0.4	T3	N1	M0	3a	3A	III	200	10	Death
**E05A0279**	56	Male	49	9	4	0.4444444	T2a	N2	M0	3a	3A	II	36	17	Death
**E05A0280**	95.2	Male	73	13	3	0.2307692	T2b	N1	M0	2b	2B	II	86.625	33	Death
**E05A0289**	63.33333	Male	75	7	1	0.1428571	T2a	N1	M0	2b	2A	II	10	59	Death
**E05A0310**	77.6	Female	69	4	1	0.25	T2a	Nx	M0	2-3	2-3	I-II	18	48	Death

Supplementary Table 2 NSCLC tissues array and detail pathological information

Number	H-score	Gender	Age	Totol LM	Posotive LN	LNM Rate	T	N	M	TNM stage	AJCC stage, 7th edition	Pathological grade	Tumor size (cm^3)	Survival time (Month)	Survival status
**E05A0317**	87.8	Female	57	12	10	0.8333333	T4	N2	M0	3b	3B	II-III	60	3	Death
**E05A0318**	90.4	Male	75	9	0	0	T4	N0	M0	3a	3A	II	48	27	Death
**E05A0321**	66.33333	Female	52	14	12	0.8571429	T3	N3	M0	3c	3B	II-III	5	44	Death
**E05A0332**	67.8	Male	55	4	0	0	T2a	N0	M0	1b	1B	II-III	49	87	Alive
**E05A0333**	75	Male	65	9	2	0.2222222	T2a	N1	M0	2b	2A	II	6	25	Death
**E05A0347**	66.4	Male	74	12	0	0	T2a	N0	M0	1b	1B	II	18.75	56	Death
**E05A0349**	54.8	Male	60	1	0	0	T2a	N0	M0	1b	1B	II	61.25	67	Death
**E05A0353**	101.75	Female	51	3	3	1	T1a	N2	M0	3a	3A	II	6	29	Death
**E05A0355**	66.6	Male	53	8	5	0.625	T2a	N2	M0	3a	3A	II	4	16	Death
**E05A0359**	88.8	Male	66	12	3	0.25	T2a	N1	M0	2b	2A	III	20	6	Death
**E05A0364**	77.4	Female	65	9	0	0	T2a	N0	M0	1b	1B	II	18	14	Death
**E05A0369**	50.25	Male	42	8	1	0.125	T1b	Nx	M0	2-3	2-3	II	12.5	13	Death
**E05A0392**	50.6	Female	57	4	2	0.5	T2a	Nx	M0	2-3	2-3	II	31.5	40	Death
**E05A0396**	53.2	Female	71	14	0	0	T1b	N0	M0	1a2	1A	II	9	84	Alive
**E05A0399**	60.2	Female	51	13	6	0.4615385	T2a	Nx	M0	2-3	2-3	II-III	36	57	Death
**E05A0403**	108.4	Male	64	7	2	0.2857143	T2a	N1	M0	2b	2A	II-III	12.5	83	Alive
**E05A0426**	66.75	Male	71	9	4	0.4444444	T3	N1	M0	3a	3A	II	36	33	Death
**E05A0448**	66.4	Female	60	16	8	0.5	T3	N3	M0	3c	3B	II	510	40	Death
**E05A0449**	85	Male	61	10	9	0.9	T2b	N3	M0	3b	3B	II-III	214.5	15	Death

Supplementary Table 2 NSCLC tissues array and detail pathological information

Number	H-score	Gender	Age	Totol LM	Posotive LN	LNM Rate	T	N	M	TNM stage	AJCC stage, 7th edition	Pathological grade	Tumor size (cm^3)	Survival time (Month)	Survival status
**E05A0450**	57.4	Female	58	6	0	0	T3	N0	M0	2b	2B	I-II	12.5	80	Alive
**E05A0456**	86	Female	62	11	9	0.8181818	T1a	Nx	M0	2-3	2-3	I-II/II-III	6	72	Death
**E05A0463**	57.2	Female	58	8	0	0	T3	N0	M0	2b	2B	I	560	35	Death
**E05A0464**	84.2	Male	60	7	1	0.1428571	T3	N1	M0	3a	3A	II	270	79	Alive
**E05A0471**	65.6	Male	63	10	3	0.3	T3	N2	M0	3b	3A	II	48	25	Death
**E05A0475**	58.5	Male	63	3	3	1	T2a	N1	M0	2b	2A	II	7.2	49	Death
**E05A0482**	63.5	Male	61	10	0	0	T2a	N0	M0	1b	1B	II-III	2	39	Death
**E05A0486**	85.4	Female	81	1	1	1	T2a	N1	M0	2b	2A	II	47.25	58	Death
**E05A0502**	83.6	Male	61	17	0	0	T2b	N0	M0	2a	2A	III	165.416	7	Death
**E05A0519**	69.75	Male	65	15	6	0.4	T2a	N2	M0	3a	3A	II-III	48	75	Alive
**E05A0520**	62	Male	74			#DIV/0!	T1a	——	M0	1a	1a	II	2	1	Death
**E05A0523**	54.2	Male	64	2	1	0.5	T2a	N1	M0	2b	2A	II	49	74	Alive
**E05A0537**	67.8	Male	53	13	0	0	T2a	N0	M0	1b	1B	III	36	52	Death
**E05A0546**	63.6	Female	73	5	4	0.8	T4	N2	M0	3b	3B	II	26.25	54	Death
**E05A0550**	77.8	Male	52	6	6	1	T2a	N2	M0	3a	3A	II	36.75	14	Death
**E05A0565**	54.8	Male	55	19	7	0.3684211	T2b	N2	M0	3b	3A	II-III	168	12	Death
**E05A0575**	76.4	Female	73	14	10	0.7142857	T2b	Nx	M0	2-3	2-3	II	157.5	12	Death
**E05A0580**	99.6	Male	44	6	1	0.1666667	T3	Nx	M0	2-3	2-3	II	180	3	Death
**E05A0584**	68	Female	50	3	0	0	T1a	N0	M0	1a2	1A	II	2.25	15	Death

Supplementary Table 2 NSCLC tissues array and detail pathological information

Number	H-score	Gender	Age	Totol LM	Posotive LN	LNM Rate	T	N	M	TNM stage	AJCC stage, 7th edition	Pathological grade	Tumor size (cm^3)	Survival time (Month)	Survival status
**E05A0592**	39.8	Female	60	21	0	0	T2b	N0	M0	2a	2A	II	160.875	2	Death
**E05A0594**	69.6	Male	54	3	3	1	T4	N2	M0	3b	3B	II-III	96	2	Death
**E05A0595**	59.75	Female	54	7	2	0.2857143	T3	N1	M0	3a	3A	II	7.5	29	Death
**E05A0596**	68.6	Female	48	14	0	0	T3	N0	M0	2b	2B	II	24.5	68	Alive
**E05A0616**	86.4	Male	59	13	10	0.7692308	T2a	N3	M0	3b	3B	III	26.25	2	Death
**E05A0622**	52	Male	78	6	0	0	T1b	N0	M0	1a2	1A	II	6.3	39	Death
**E05A0627**	75.4	Male	59	38	21	0.5526316	T2a	Nx	M0	2-3	2-3	II	48	25	Death
**E05A0633**	82.33333	Male	58	28	0	0	T2a	N0	M0	1b	1B	III	72	68	Alive
**E05A0639**	75	Female	56	9	4	0.4444444	T4	N2	M0	3b	3B	II	31.5	15	Death
**E05A0640**	63.2	Female	53	4	0	0	T2a	N0	M0	1b	1B	I-II	61.25	67	Alive
**E05A0648**	85	Female	62	6	1	0.1666667	T1b	Nx	M0	2-3	2-3	II	15	10	Death
**E05A0659**	63.4	Male	72	17	11	0.6470588	T2a	N2	M0	3a	3A	II	40	30	Death
**E05A0663**	61.66667	Male	84	9	0	0	T2b	N0	M0	2a	2A	I-II	110	24	Death
**E05A0671**	53.8	Male	61	19	0	0	T2b	N0	M0	2a	2A	II-III	137.5	65	Alive
**E05A0677**	77.8	Female	67	19	10	0.5263158	T1b	Nx	M0	2-3	2-3	II	12.5	64	Alive
**E05A0682**	56	Female	65	5	0	0	T2a	N0	M0	1b	1B	II	36	55	Death
**E05A0699**	89.8	Male	65	11	2	0.1818182	T3	N2	M0	3b	3A	III	84	8	Death
**E05A0702**	66.2	Male	65	15	0	0	T3	N0	M0	2b	2B	III	96.25	64	Alive
**E05A0703**	70.6	Female	77	12	8	0.6666667	T3	N3	M0	3c	3B	II	12.5	29	Death

Supplementary Table 2 NSCLC tissues array and detail pathological information

Number	H-score	Gender	Age	Totol LM	Posotive LN	LNM Rate	T	N	M	TNM stage	AJCC stage, 7th edition	Pathological grade	Tumor size (cm^3)	Survival time (Month)	Survival status
**E05A0721**	70.6	Male	50	3	0	0	T2a	N0	M0	1b	1B	III	36		lost
**E05A0726**	67.8	Female	66	5	0	0	T2a	N0	M0	1a1	1B	I	15	62	Alive

Supplementary Table 3 KEGG Enrichment: A549 NADK VS. A549 Control

Direction	GSEA analysis: NA vs EV	NES	Genes	adj.Pval
Down	TNF signaling pathway	-2.6055	86	1.3e-02
	NF-kappa B signaling pathway	-2.5939	70	1.3e-02
	Legionellosis	-2.5621	34	3.2e-02
	Rheumatoid arthritis	-2.4935	39	5.2e-02
	IL-17 signaling pathway	-2.489	57	5.2e-02
	Epithelial cell signaling in Helicobacter pylori infection	-2.4637	54	6.0e-02
	Viral protein interaction with cytokine and cytokine receptor	-2.4088	20	3.3e-02
	NOD-like receptor signaling pathway	-2.382	107	3.5e-02
	Kaposi sarcoma-associated herpesvirus infection	-2.3437	125	3.3e-02
	Cytokine-cytokine receptor interaction	-2.3315	86	6.7e-02
	Lipid and atherosclerosis	-2.2154	130	6.4e-02
	RIG-I-like receptor signaling pathway	-2.214	40	7.1e-02
	Influenza A	-2.1936	93	8.6e-02
	Epstein-Barr virus infection	-2.1916	131	6.7e-02
	Pertussis	-2.1795	39	7.1e-02
	C-type lectin receptor signaling pathway	-2.1734	65	8.6e-02
	Osteoclast differentiation	-2.143	71	8.6e-02
	Toll-like receptor signaling pathway	-2.1227	55	8.6e-02
	Measles	-2.1203	84	8.9e-02
	Tuberculosis	-2.0981	98	9.1e-02
	Leishmaniasis	-2.0952	34	7.0e-02
	Chagas disease	-2.0548	65	8.6e-02
	Amoebiasis	-1.9788	58	8.6e-02
	Chemokine signaling pathway	-1.9578	101	9.7e-02
	Ferroptosis	-1.9467	32	7.0e-02
	Hepatitis C	-1.9376	103	9.7e-02
	Small cell lung cancer	-1.9366	79	8.8e-02
	Necroptosis	-1.9332	91	9.1e-02
	Apoptosis	-1.9173	109	9.9e-02
	Viral carcinogenesis	-1.8962	124	1.0e-01
	Phagosome	-1.8782	77	8.7e-02

Supplementary Table 3 KEGG Enrichment: A549 NADK VS. A549 Control

Direction	GSEA analysis: NA vs EV	NES	Genes	adj.Pval
	Alcoholic liver disease	-1.8758	91	9.1e-02
	T cell receptor signaling pathway	-1.8295	63	8.6e-02
	Transcriptional misregulation in cancer	-1.8214	117	1.0e-01
	PD-L1 expression and PD-1 checkpoint pathway in cancer	-1.8166	59	8.6e-02
	Toxoplasmosis	-1.7731	65	8.7e-02
	Yersinia infection	-1.7655	101	9.7e-02
	Proteasome	-1.7554	35	8.6e-02
	Glycerophospholipid metabolism	-1.7456	64	8.7e-02
	Acute myeloid leukemia	-1.7234	50	8.7e-02
Up	GABAergic synapse	1.878	36	3.4e-02
	Morphine addiction	1.8565	42	3.4e-02
	Gastric acid secretion	1.8313	38	4.0e-02
	Taste transduction	1.8016	16	6.0e-02
	Calcium signaling pathway	1.7919	105	1.3e-02
	Salivary secretion	1.7721	39	5.7e-02
	Olfactory transduction	1.7703	16	6.7e-02
	Bile secretion	1.7288	31	6.7e-02
	TGF-beta signaling pathway	1.7246	68	4.0e-02
	Dilated cardiomyopathy	1.7176	50	5.8e-02
